# Biofilm Formation Potential of Heat-Resistant Escherichia coli Dairy Isolates and the Complete Genome of Multidrug-Resistant, Heat-Resistant Strain FAM21845

**DOI:** 10.1128/AEM.00628-17

**Published:** 2017-07-17

**Authors:** Roger Marti, Michael Schmid, Sandra Kulli, Kerstin Schneeberger, Javorka Naskova, Susanne Knøchel, Christian H. Ahrens, Jörg Hummerjohann

**Affiliations:** a Agroscope, Division of Food Microbial Systems, Microbiological Safety of Foods of Animal Origin Group, Bern, Switzerland; b Agroscope, Research Group Molecular Diagnostics, Genomics and Bioinformatics, and SIB Swiss Institute of Bioinformatics, Wädenswil, Switzerland; c Department of Food Science, University of Copenhagen, Copenhagen, Denmark; University of Helsinki

**Keywords:** biofilm, locus of heat resistance, LHR, Escherichia coli, persistence, antimicrobial resistance, dairy, biofilms

## Abstract

We tested the biofilm formation potential of 30 heat-resistant and 6 heat-sensitive Escherichia coli dairy isolates. Production of curli and cellulose, static biofilm formation on polystyrene (PS) and stainless steel surfaces, biofilm formation under dynamic conditions (Bioflux), and initial adhesion rates (IAR) were evaluated. Biofilm formation varied greatly between strains, media, and assays. Our results highlight the importance of the experimental setup in determining biofilm formation under conditions of interest, as correlation between different assays was often not a given. The heat-resistant, multidrug-resistant (MDR) strain FAM21845 showed the strongest biofilm formation on PS and the highest IAR and was the only strain that formed significant biofilms on stainless steel under conditions relevant to the dairy industry, and it was therefore fully sequenced. Its chromosome is 4.9 Mb long, and it harbors a total of five plasmids (147.2, 54.2, 5.8, 2.5, and 1.9 kb). The strain carries a broad range of genes relevant to antimicrobial resistance and biofilm formation, including some on its two large conjugative plasmids, as demonstrated in plate mating assays.

**IMPORTANCE** In biofilms, cells are embedded in an extracellular matrix that protects them from stresses, such as UV radiation, osmotic shock, desiccation, antibiotics, and predation. Biofilm formation is a major bacterial persistence factor of great concern in the clinic and the food industry. Many tested strains formed strong biofilms, and especially strains such as the heat-resistant, MDR strain FAM21845 may pose a serious issue for food production. Strong biofilm formation combined with diverse resistances (some encoded on conjugative plasmids) may allow for increased persistence, coselection, and possible transfer of these resistance factors. Horizontal gene transfer may conceivably occur in the food production setting or the gastrointestinal tract after consumption.

## INTRODUCTION

As opposed to the planktonic cultures normally used in laboratory settings, the predominant bacterial mode of growth in nature is as surface-adherent communities called biofilms. Bacteria growing in biofilms are embedded in a matrix mainly consisting of extracellular polymeric substances. The exact composition of the matrix depends on the bacteria comprising the biofilm, but the major constituents are proteins, nucleic acids, polysaccharides, lipids, and water (1). The biofilm lifestyle protects the bacterial community from environmental stresses, such as UV radiation, osmotic shock, desiccation, antibiotics (up to 1,000-fold increased resistance), predation by invertebrates, and the (human) immune system (2, 3). The metabolic activities of cells in a biofilm vary greatly, with a dormant subset, so-called persister cells, being very difficult to eradicate due to their low metabolic activity (4). For these reasons, biofilms are a major concern not only in clinical settings, where they cause wound infections or colonize medical devices (5), but also in the food industry. Formation of biofilms on rubber, polyethylene, stainless steel, glass, and other food contact surfaces can severely complicate cleaning procedures, which may lead to outbreaks of foodborne illness (6–8).

Heat treatment is a widely used method for inactivation of microbes. While autoclaving ensures killing of even bacterial spores, it is often not a feasible option in both clinical and food industry settings, and milder treatment is needed. For this reason, even moderate heat resistance, i.e., resistance that is much lower than that of spores, is a major concern with regard to decontamination and bacterial persistence and can have dire consequences. Thermization of raw milk at subpasteurization temperatures is used to increase safety while leaving the natural microbiota and enzymes intact (9). There is also a general trend in consumer behavior of preferring minimally processed food, and there are foods (i.e., meats and fresh produce) that simply cannot be heated sufficiently to reliably kill all pathogens without severe degradation of nutrients (10). This leads to a balancing act between food safety, consumer desire, sensory properties, and nutritional quality of food. In clinical settings, flexible endoscopes are a prime example of a complex tool (with long, narrow channels where bacteria may form biofilms and remain) which usually cannot be treated at high temperatures and for which other decontamination procedures are required (11). A thermochemical treatment (<60°C) was ineffective at eradicating a Klebsiella pneumoniae isolate which harbored a plasmid-borne operon encoding a novel Clp ATPase, ClpK, mediating increased heat resistance (12). Only 1 year later, close homologs of much of the operon, including *clpK*, were found on the chromosome of Cronobacter sakazakii ATCC 29544, and an extensive PCR screening (for *clpK* and another marker gene, *orfI*) found similar sequences in other Enterobacteriaceae, including Escherichia coli (13). Comparative genetic analysis found *clpK* and its surrounding region (flanked by mobile elements; ∼14 kb), now termed the locus of heat resistance (LHR), in approximately 2% of available E. coli whole-genome (shotgun) sequences, including those of pathogens and food isolates (14). Several heat-resistant strains were found in the course of *in vitro* characterization of E. coli raw milk isolates (15). These strains were further analyzed, and the results confirmed increased resistance to subpasteurization temperatures in milk (16) as well as boosted survival during ripening of semihard raw milk cheese (17). In a recent study, 256 E. coli raw milk cheese isolates were screened by PCR for *clpK* (12) and *orfI* (13). Ninety-three (36.3%) of these isolates tested positive for both marker genes, while 24 and 9, respectively, gave single-positive results. We speculated that a thermal selection pressure might have caused this increased abundance compared to the above-mentioned estimated 2% among all E. coli strains. In addition, double-positive strains were phenotypically heat resistant in 95.7% of cases, confirming the great predictive accuracy of these PCRs (18). In contrast, a very recent quantitative PCR (qPCR) study found the LHR in only 0.5% of clinical E. coli isolates (*n* = 613) (19), which further supports the speculation of a selective pressure in the dairy environment. For the purpose of this study, heat resistance refers to that mediated by *clpK* and the LHR only.

A combination of both strong biofilm formation potential and heat resistance can further exacerbate the problem of bacterial persistence. Biofilm formation can further protect heat-resistant K. pneumoniae from heat shock (20). Also, heat-resistant, extended-spectrum β-lactamase (ESBL)-harboring K. pneumoniae recently caused a nosocomial outbreak (21). Persistent intramammary E. coli infections in dairy cows are likely due to biofilm formation of the infecting strains (22), and biofilms can be found on many contact surfaces in the food industry (23). Should such strains be heat resistant as well, increased contamination of raw milk products is to be expected (16, 17). This may then lead to an increased incidence of early blowing of cheese (24) or to recalls due to exceeding the limits on coliform CFU set by hygienic standards. We have not yet isolated pathogenic, heat-resistant E. coli strains from dairy products. However, our previous studies demonstrated the possibility of transfer of Shiga toxin-encoding phage and ESBL plasmids to heat-resistant E. coli (18) and of both LHR of the heat-resistant dairy isolate FAM21805 to other E. coli strains, including pathogenic strains (25). Because biofilm formation typically increases rates of horizontal gene transfer (HGT) (26), it is also of concern in this context.

We evaluated the biofilm formation potential of 30 heat-resistant and 6 heat-sensitive E. coli strains isolated from raw milk and raw milk cheese, as well as that of E. coli K-12 MG1655, which was used as a reference. A large number of phenotypic/functional assays were used to determine biofilm formation under both static (crystal violet [CV] assays in 96-well polystyrene [PS] plates and on stainless steel coupons [SSC]) and dynamic (initial adhesion rate [IAR] and Bioflux flow cell [FC] assays) conditions. Macrocolony assays were employed to qualitatively assess production of curli and cellulose, two major components of the E. coli biofilm matrix. The strongest overall biofilm former under static conditions and the only isolate able to form biofilm on SSC was FAM21845. We therefore fully sequenced and assembled the genome of this isolate. It was analyzed with respect to its LHR and biofilm formation genes and was also found to carry multiple antimicrobial resistance (AMR) and heavy metal resistance genes. We detected the presence of five plasmids, including one harboring several AMR genes, a heavy metal resistance operon, and a disinfectant resistance gene and one containing a TEM-1 β-lactamase gene and the *mrkABCDF* operon (encoding type III fimbriae), known to increase biofilm formation. Conjugation of the *mrk*-containing plasmid into K-12 MG1655 resulted in more biofilm production, underlining the potential threat of strains such as FAM21845 in the food industry. The combination of these many resistance and persistence factors is problematic, as coselection can lead to retention of all of them, even if selection pressure is applied to only one. Strains such as FAM21845 give rise to another concern: the possible spread of resistance and persistence factors in the food industry. Also, transfer of antimicrobial resistance genes in the gastrointestinal tract of humans and in animal models has been observed (reviewed in references 27 and 28), and the possibility of spread via this route cannot be excluded.

## RESULTS

### All strains have phenotypically active *rpoS*.

Overall, we tested 37 E. coli strains for their potential biofilm formation under a variety of conditions in this study. These included 30 heat-resistant and 6 heat-sensitive strains isolated from raw milk and raw milk cheese, as well as E. coli K-12 MG1655, used as a further heat-sensitive strain and a reference. Strains were considered heat resistant if their reduction in CFU after 30 min of incubation at 55°C was less than 1 log. Notably, 2 of the heat-sensitive isolates are ESBL producers (Table 1). Because biofilm formation of E. coli is influenced by the stationary-phase sigma factor encoded by *rpoS* (29), we first tested the activity of catalase as an indirect method to confirm RpoS-mediated transcription (30, 31). All 37 strains tested positive for catalase activity and should in turn encode functional, active RpoS (30, 31).

**TABLE 1:** E. coli strains used in this study

Strain	Serovar	Phylogenetic group^*a*^	Sequence type^*b*^	Characteristic(s)^*c*^	Antibiotic resistance profile^*d*^	Dairy origin	Reference(s)
FAM19195	O8:H21	B1		Heat resistant	str	Raw milk cheese	15, 16
FAM21805	O68:H14	A		Heat resistant		Raw milk cheese	15, 16
FAM21807	O68:H14	A		Heat resistant	str	Raw milk cheese	15
FAM21808	O11:H11	A		Heat resistant	str	Raw milk cheese	15
FAM21843	O178:H12	A		Heat resistant	STR, TMP	Raw milk cheese	15, 16
FAM21845	O68:H14	A	ST1434	Heat resistant	GEN, KAN, STR, TET, TMP, SXT, AMP	Raw milk cheese	15
FAM22636		A		Heat resistant	str	Raw milk cheese	18
FAM22639		A		Heat resistant	str	Raw milk cheese	18
FAM22791		A		Heat resistant		Raw milk cheese	18
FAM22808		A		Heat resistant		Raw milk cheese	18
FAM22891		B1		Heat resistant	GEN, KAN, STR, CHL, TET, TMP, SXT, AMP	Raw milk cheese	18
FAM22936		A		Heat resistant	str	Raw milk cheese	18
FAM22940		B1		Heat resistant	amp, cef	Raw milk cheese	18
FAM22947		A		Heat resistant		Raw milk cheese	18
FAM22954		A		Heat resistant		Raw milk cheese	18
FAM22961		A		Heat resistant		Raw milk cheese	18
FAM22962		A		Heat resistant		Raw milk cheese	18
FAM22963		A		Heat resistant		Raw milk cheese	18
FAM23012		A		Heat resistant	TET	Raw milk cheese	18
FAM23014		A		Heat resistant	str	Raw milk cheese	18
FAM23016		A		Heat resistant	str	Raw milk cheese	18
FAM23030		A		Heat resistant		Raw milk cheese	18
FAM23031		A		Heat resistant	str	Raw milk cheese	18
FAM23078		A		Heat resistant	str	Raw milk cheese	18
FAM23092		A		Heat resistant	TET	Raw milk cheese	18
FAM23093		A		Heat resistant	TET	Raw milk cheese	18
FAM23101		A		Heat resistant	GEN, KAN, TET, TMP, SXT, AMP	Raw milk cheese	18
FAM23106		A		Heat resistant	str	Raw milk cheese	18
FAM23109		A		Heat resistant	STR, TET, AMP	Raw milk cheese	18
FAM23113		A		Heat resistant	KAN, STR, CHL, TET, AMP, cef, amc	Raw milk cheese	18
FAM21846	O16:H21	A		Heat sensitive		Raw milk cheese	Present study
FAM22942		B1		Heat sensitive	STR, CHL, TET, AMP, cef	Raw milk cheese	Present study
FAM22956		B1		Heat sensitive	str	Raw milk cheese	Present study
FAM22996		A		Heat sensitive	TET, AMP	Raw milk cheese	Present study
FAM22321		A	ST4483	Heat-sensitive, ESBL phenotype; TEM-1, CTX-M-14	GEN, KAN, STR, CHL, TET, NAL, CIP, TMP, SXT, AMP, CEF, CXM, CTX, atm	Raw milk	18
FAM22871		E	ST69, STC69	Heat-sensitive, ESBL phenotype; TEM-1, CTX-M-15	GEN, KAN, STR, TET, AMP, CEF, CXM, CTX, ATM	Raw milk	18
K-12 MG1655				Heat sensitive; DSM 18039			
K-12 MG1655 NAL^r^ RIF^r^				Heat sensitive; spontaneous resistant mutant	NAL, RIF		95

a Determined by quadruplex and group C- and E-specific PCRs (76).

b Determined by use of a 7-allele multilocus sequence typing scheme (MLST Database at UoW) (77).

c All strains tested positive for catalase activity. Heat resistant, *clpK* and *orfI* positive by PCR and phenotypically heat resistant (<1-log reduction in CFU after 30 min of incubation at 55°C) (18).

d Antimicrobial resistances were determined according to CLSI guidelines (78). Capital and lowercase letters indicate resistant and intermediate phenotypes, respectively. Antimicrobials tested were gentamicin (GEN), kanamycin (KAN), streptomycin (STR), chloramphenicol (CHL), tetracycline (TET), nalidixic acid (NAL), ciprofloxacin (CIP), trimethoprim (TMP), sulfamethoxazole-trimethoprim (19:1) (SXT), ampicillin (AMP), cefoxitin (FOX), cephalothin (CEF), cefuroxime (CXM), cefotaxime (CTX), cefepime (FEP), aztreonam (ATM), amoxicillin-clavulanic acid (20:10) (AMC), and ertapenem (ETP).

### Curli and cellulose production.

Amyloid curli fibers and cellulose are two major components of the E. coli biofilm extracellular matrix (32). Macrocolonies are a form of biofilm and assume structured morphologies dependent on the production of these two matrix constituents. Production of both curli fibers (curli) and cellulose results in highly structured colonies with a network-like appearance, while large amounts of curli without cellulose result in colonies with concentric wrinkled rings (33). Congo red (staining both curli and cellulose [34]) and calcofluor (staining cellulose [35]) further aid in the qualitative evaluation of production of these two matrix components. In most but not all E. coli strains, the regulator CsgD, mediating expression of both curli and cellulose, is expressed at temperatures below 30°C (35). It was found that for K-12 strains, CsgD production is higher, resulting in more curli and cellulose production, when these strains are grown on salt-free LB (LBnoS) plates (36). In general, the combination of a temperature below 30°C and salt-free medium is expected to result in the strongest production of both curli and cellulose (37). We grew macrocolonies on regular and salt-free LB agar plates (in addition to AB minimal medium with 0.5% Casamino Acids [ABTCAA] and diluted reconstituted powdered skim milk [RPSM_dil_] agars) at both 28 and 37°C to assess the impacts of salt and temperature on curli and cellulose production in the present strains.

**FIG 1:**
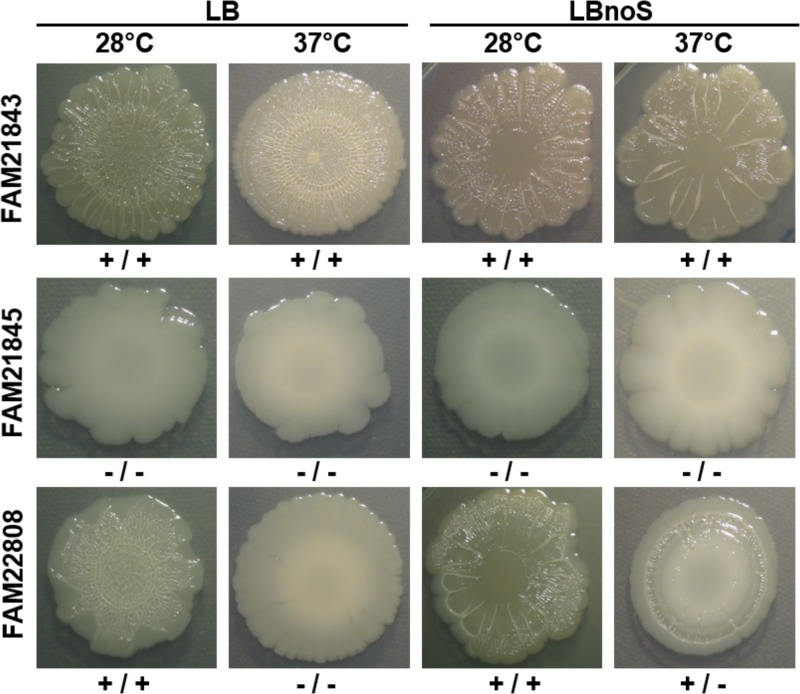
Examples of macrocolony assays. Seven-day macrocolonies incubated on LB or LBnoS at either 28 or 37°C showed different expression patterns of curli fibers and cellulose. FAM21843 produced both matrix constituents under all conditions tested, FAM21845 was unable to produce either, and FAM22808 produced both but strongly reacted to changes in salt concentration and temperature (see Fig. S1 in the supplemental material for a complete set of macrocolony images). The first plus or minus symbol beneath each image represents the presence or absence of curli, and the second represents the presence or absence of cellulose.

Images of macrocolonies for all 37 strains at 28 and 37°C for all media tested in this study are given in Fig. S1 in the supplemental material. We found three main behaviors regarding curli and cellulose expression at 28°C versus 37°C and with salt-containing versus salt-free LB: (i) double-positive strains with no changes, (ii) double-negative strains with no changes, and (iii) double-positive strains with strong changes due to temperature and salt content (Fig. 1). LBnoS at 28°C resulted in the most curli/cellulose double-positive macrocolonies (9 colonies). This number was reduced to 5 at 37°C. For LB, this number was 8 at 28°C and 2 at 37°C, and for ABTCAA, it was 7 at 28°C and 1 at 37°C. Neither curli nor cellulose was produced on RPSM_dil_ agar at either temperature by any strain tested (Table S1). The switch from 28 to 37°C reduced production of curli and/or cellulose in 7 isolates on LBnoS, 15 on LB, and 12 on ABTCAA. FAM21843 was the only strain which produced both matrix constituents on all media at both temperatures. The production of curli and cellulose showed significant correlations for all media and temperatures tested, with the lowest correlation coefficient (0.478) observed for ABTCAA at 37°C (Table S2).

### Biofilm formation on polystyrene surfaces.

We tested all 37 E. coli strains for biofilm formation on PS surfaces in LB, LBnoS, RPSM_dil_, and ABTCAA media at 12, 28, and 37°C by using CV assays. A clear dependency of the extent of biofilm formation on both medium and temperature was observed (Table 2). For each medium except RPSM_dil_, 28°C led to the greatest overall score for biofilm formation. RPSM_dil_ also showed the lowest overall score, by far, at 12°C. In contrast to the other three media, there was no strain which did not form a biofilm in RPSM_dil_. It is also interesting that 37°C, while being the temperature with the lowest overall biofilm formation for the rich media LB and LBnoS, was the best and second best temperature for RPSM_dil_ and ABTCAA, respectively. The best overall medium for biofilm formation at each individual temperature tested was ABTCAA.

**TABLE 2:** Static biofilm formation on PS and SSC after 48 h of incubation and biofilm formation in FC channel after 24 h^*a*^

Strain	Characteristic(s)	OD/ODc category for 96-well PS plates^*b*^												Overall score	OD/ODc category for SSC^*b*^	Biofilm formation in FC channel^*c*^
		LB			LBnoS			RPSM_dil_			ABTCAA				RPSM	ABTCAA
		12°C	28°C	37°C	12°C	28°C	37°C	12°C	28°C	37°C	12°C	28°C	37°C		12°C	37°C
FAM19195	HR	−	4	−	−	3	−	−	3	3	1	4	2	20	−	−
FAM21805	HR	1	4	2	−	3	1	−	2	3	1	3	3	23	−	+
FAM21807	HR	1	3	−	−	2	1	−	3	3	1	3	2	19	−	−
FAM21808	HR	−	−	−	−	−	−	−	3	3	−	1	1	8	−	−
FAM21843	HR	1	4	2	1	4	1	−	3	3	4	4	3	30	−	+
FAM21845	HR	4	3	3	4	4	3	2	4	3	4	4	4	42	1	−
FAM22636	HR	1	3	1	1	2	1	−	2	2	1	2	3	19	−	−
FAM22639	HR	3	2	2	3	1	2	−	3	2	2	3	3	26	−	−
FAM22791	HR	2	2	2	2	2	2	−	3	3	1	2	2	23	−	−
FAM22808	HR	1	4	1	−	4	1	−	3	3	2	3	2	24	−	−
FAM22891	HR	3	2	1	3	2	1	−	3	3	3	2	4	27	−	−
FAM22936	HR	4	2	2	4	1	1	−	2	2	3	3	3	27	−	+
FAM22940	HR	−	1	1	−	−	−	−	3	3	1	2	2	13	−	−
FAM22947	HR	2	1	2	1	1	2	−	4	4	2	3	2	24	−	−
FAM22954	HR	2	4	2	2	4	2	1	3	3	3	4	4	34	−	−
FAM22961	HR	2	3	1	2	3	1	1	3	3	3	4	2	28	−	−
FAM22962	HR	−	−	−	−	−	−	−	3	2	−	4	2	11	−	+
FAM22963	HR	3	3	1	3	3	2	1	3	3	4	4	2	32	−	−
FAM23012	HR	4	3	3	3	2	2	−	2	2	3	3	4	31	−	+
FAM23014	HR	3	3	2	3	2	2	−	3	3	3	3	3	30	−	−
FAM23016	HR	3	3	2	3	4	2	1	2	2	4	4	3	33	−	−
FAM23030	HR	2	3	1	3	4	1	1	2	3	4	4	2	30	−	−
FAM23031	HR	1	1	1	1	1	1	−	2	3	1	3	1	16	−	−
FAM23078	HR	−	1	1	−	1	−	−	2	2	1	4	3	15	−	−
FAM23092	HR	3	3	3	3	2	3	−	2	2	2	3	4	30	−	+
FAM23093	HR	4	3	2	3	3	2	−	1	2	2	3	3	28	−	+
FAM23101	HR	3	3	2	4	3	2	−	3	2	4	3	4	33	−	+
FAM23106	HR	−	1	−	−	1	−	−	2	2	1	4	3	14	−	−
FAM23109	HR	1	2	2	2	2	2	1	2	2	3	4	3	26	−	−
FAM23113	HR	3	3	2	4	2	2	−	2	3	3	3	3	30	−	−
FAM21846	HS	−	1	1	−	3	1	−	−	2	1	3	3	15	−	−
FAM22942	HS	1	4	4	1	4	3	−	2	2	2	4	4	31	−	+
FAM22956	HS	1	4	4	1	3	4	−	3	3	2	3	3	31	−	−
FAM22996	HS	−	−	−	−	−	−	−	1	3	−	−	−	4	−	−
FAM22321	HS, ESBL	−	1	−	1	1	1	−	2	3	−	−	1	10	−	−
FAM22871	HS, ESBL	1	4	1	1	4	1	−	2	1	1	4	2	22	−	+
K-12 MG1655	HS	1	4	1	1	4	1	−	2	2	2	4	2	24	−	+
Overall score		61	92	55	60	85	51	8	90	95	75	114	97		1 positive	11 positives

a LB, Luria-Bertani Lennox broth; LBnoS, LB without addition of NaCl; RPSM and RPSM_dil_, reconstituted powdered skim milk at 10.5% and 0.2% (wt/vol), respectively; ABTCAA, AB minimal medium with 0.5% Casamino Acids as a carbon source (56); HR, heat resistant; HS, heat sensitive; ESBL, extended-spectrum β-lactamase; FC, Bioflux flow cells; SSC, stainless steel coupons.

b Numbers in columns indicate categories defined by OD/ODc ratios (79). The cutoff value was determined as follows: ODc = average OD_negative control_ + 3× SD(OD_negative control_). The categories are indicated as follows: −, OD ≤ ODc; 1, ODc < OD ≤ 2× ODc; 2, 2× ODc < OD ≤ 4× ODc; 3, 4× ODc < OD ≤ 8× ODc; and 4, 8× ODc < OD.

c +, formation of biofilm within the FC channel for each replicate.

As with media and temperatures, there were large variations in biofilm formation between strains. Overall scores per strain ranged from 4 to 42, with K-12 MG1655 falling in the midrange (score of 24). Only seven strains had scores of ≥1 under all conditions tested (FAM21845, FAM22954, FAM22961, FAM22963, FAM23016, FAM23030, and FAM23109) (Table 2). The variability of biofilm formation (measured by the standard deviation [SD] of all category values) was twice as high for FAM19195 (1.60) as for FAM21845 (0.65), demonstrating clear differences in the extent of biofilm formation regulation under the conditions tested. Some strains, such as FAM22996, were consistent in their lack of biofilm formation over almost all media and temperatures tested (overall score = 4; SD = 0.85).

Strains with overall low biofilm formation scores tended to form very little to no biofilm in LB and LBnoS. Some weak biofilm formers (FAM22996, FAM21808, and FAM22321) also formed little or no biofilm in ABTCAA (Table 2). FAM21845 had the highest overall score (42; the second highest was 34, for FAM22954), being the most consistent biofilm former (Table 2), although it did not always have the highest absolute optical density/cutoff optical density (OD/ODc) ratio. At 28°C, we found positive correlations between cellulose production and CV scores for all media, and also between curli production and CV scores for ABTCAA (Table S2).

### Initial adhesion rates on polyvinyl chloride (PVC).

**FIG 2:**
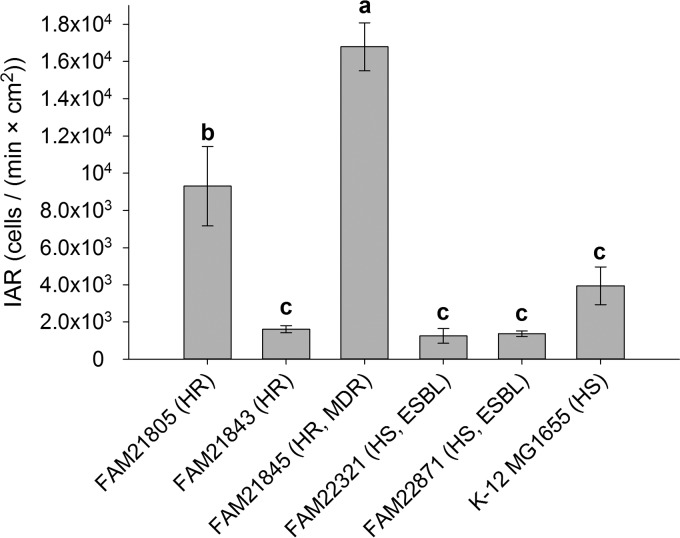
Initial adhesion rates (IAR) of six E. coli strains. IAR of two heat-resistant (HR) strains, one HR and MDR strain, two heat-sensitive (HS) ESBL producers, and K-12 MG1655 were determined. Three distinct groups of strains differing in their IAR emerged (a, b, and c). Bars indicate averages and standard deviations, and columns not sharing a letter are significantly different from one another (one-way ANOVA, all pairwise multiple comparison, Holm-Sidak test; α = 0.05).

The first step in biofilm formation is the initial adhesion of cells to a solid surface, which is then followed by maturation if conditions allow (38). To assess this critical step, we performed IAR measurements for six strains (Fig. 2). IAR varied by a factor of >10, from 1.2 × 10^3^ cells/(min · cm^2^) for FAM22321 to 1.7 × 10^4^ cells/(min · cm^2^) for FAM21845. One-way analysis of variance (ANOVA) found significant differences between three groups of strains, with FAM21845 exhibiting the highest IAR, followed by FAM21805 (Fig. 2).

### Biofilm formation under dynamic conditions.

**FIG 3:**
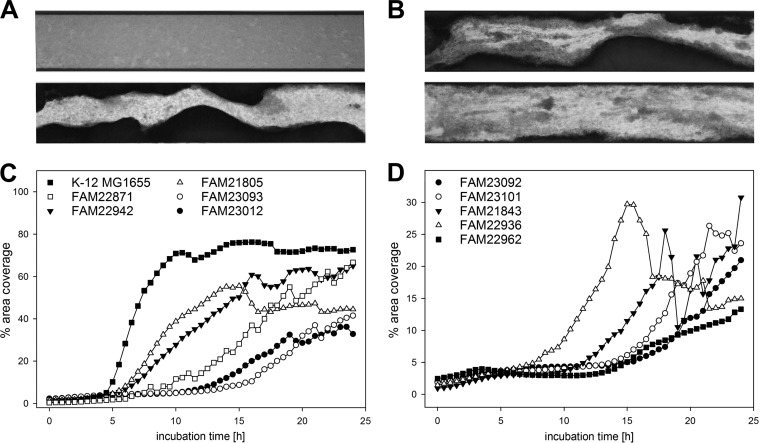
Biofilm development under dynamic conditions in flow cells (FC) of the Bioflux system. (A and B) Bottom-up views of FC (5× objective, bright field). The imaged parts of the FC were approximately 1.9 mm long and 350 μm wide (FC cross-section, 350 × 70 μm), and the medium flow was from left to right. (A) FAM19195 (19 h) showed biofilm from the sides and a bacterial lawn but no biofilm formation within the channel (top), and FAM21805 (19 h) produced biofilm from the sides and within the channel (bottom). (B) There was a sudden decrease (sloughing) of strong biofilm within the channel for strain FAM21843 between 18 h (top) and 18.5 h (bottom). (C and D) Average area coverage (% of the flow channel within an image that was covered by biofilm) was evaluated every 30 min for 24 h for all 37 strains. Eleven strains consistently formed biofilm within the FC channel, and their area coverages over time are given here. Values indicate averages for at least biological triplicates.

Biofilms may form not only under static conditions, as tested in our CV assays on PS, but also under dynamic conditions. Dynamism in the surrounding medium changes the conditions for biofilm formation by introducing shear forces and delivering fresh medium to adherent cells (no nutrient depletion) (39). To assess biofilm formation under dynamic conditions, we used the Fluxion Bioflux system. As ABTCAA was found to be the best overall medium for biofilm formation under static conditions, this medium was chosen for dynamic biofilm formation assays. For technical reasons, experiments were run for 24 instead of 48 h, and 37°C was used to allow for sufficient growth, even though the overall score was slightly higher at 28°C under static conditions (overall score of 114 versus 97). Due to variation between replicates, strains had to be categorized into two sets, with either good or poor overall reproducibility. Good reproducibility was assigned to strains that consistently either did or did not form biofilm on the sides of the channel and/or within the FC channel between replicates. Poor reproducibility was defined as biofilm formation in some but not all replicates (Table S1). All strains were able to produce biofilm on the walls of the channel (with poor reproducibility for FAM21808, FAM22954, and FAM22996). A bacterial lawn, defined as light gray coverage clearly darker than that of the negative control but much less dense than the true biofilm covering the bottom of the channel, was formed by all strains (with poor reproducibility for FAM21808, FAM23078, FAM23106, and FAM22996) (Fig. 3A). A total of 11 strains showed good reproducibility and consistently produced biofilm within the FC channel. Average area coverage graphs for these strains are given in Fig. 3C and D. Importantly, the area coverage percentage varied greatly between replicates for most strains, even if they produced biofilm within the FC channel in every replicate. For seven strains consistently forming biofilm within the FC channel (FAM21805, FAM21843, FAM22871, FAM22936, FAM23012, FAM23093, and K-12 MG1655), a sudden decrease of area coverage between two time points was observed. This sloughing-off biofilm material was observed in individual replicates only. It is illustrated for strain FAM21843 at 18 versus 18.5 h of incubation (Fig. 3B). The fastest strains reached 5% average area coverage within 4.5 h (K-12 MG1655) and 5.5 h (FAM21805 and FAM22942), while FAM23101 exceeded 5% average area coverage only after 14 h. The highest average area coverage, 76.3%, was reached by K-12 MG1655 after 16 h of incubation. FAM22962 was the weakest of the consistent biofilm formers, and its average area coverage increased until the end of the experiment (13.3% after 24 h). We note that FAM21845, the strongest, most consistent biofilm former on PS surfaces, did not form biofilm in the Bioflux FC in any replicate.

### Only FAM21845 forms biofilm on stainless steel coupons.

In a final biofilm experiment, we wanted to assess biofilm formation under conditions relevant to industry: on SSC in full-strength RPSM at 12°C. Only FAM21845 yielded an OD/ODc ratio of >1 and therefore scored a “1” in our categorization scheme (Table 2). The average ratios for the top five strains were 1.30, 0.92, 0.91, 0.87, and 0.86 for FAM21845, FAM23113, FAM23092, FAM21805, and FAM23014, respectively. The ratio for FAM21845 was significantly higher than that for all other strains taken together (Mann-Whitney rank sum test of individual replicates; *P* = 0.004). Comparing strains pairwise, biofilm formation of FAM21845 on SSC was significantly stronger than that of all other strains except FAM23113 and FAM23092 (one-tailed *t* test; *P* < 0.05).

### FAM21845 carries antimicrobial and heavy metal resistance genes.

FAM21845 showed the highest IAR and was the best biofilm former of the strains tested in this study under static conditions on PS and SSC. Assays on SSC were performed at 12°C in milk-like medium, an assay mimicking conditions encountered in the dairy/food industry. This strain is also heat resistant and multidrug resistant (MDR) (Table 1). For these reasons and to be able to link some of the observed phenotypes with the genotype, and as a basis for future functional genomics or systems biology studies (40, 41), we decided to sequence the entire genome of FAM21845.

The chromosome of FAM21845 is 4.9 Mb long, with a GC content of 51.0%, and was predicted to carry 4,812 coding DNA sequences (CDS) (Table 3). The strain belongs to sequence type ST1434 (Table 1). It carries genes for resistance against β-lactams (*ampC* and *ampH*) and 156 CDS with very high similarity (with strict criteria and manual curation) to sequences in the antibacterial biocides and metal resistance gene database BacMet (42) (Table S3). Among these are genes encoding resistance-nodulation-cell division (RND) efflux pumps (AcrAB, AcrAD, AcrEF, MdtABC, and MdtEF, each with TolC and CusCFBA) with broad substrate ranges including antimicrobials (43), as well as three operons of interest: (i) the arsenic resistance operon *ars*, composed of *arsRBC* as well as *arsRDABC*, an extended version of the operon associated with further increased arsenic resistance (44, 45); (ii) the silver resistance operon *sil*, split into two transcriptional units, *silRSE* and *silCFBAP*, including the hypothetical protein gene *orf105* between *silA* and *silP* (46); and (iii) the copper resistance operon *pcoABCDRSE* (47).

**TABLE 3:** Chromosome and plasmids of FAM21845

Genetic location	Size (bp)	% G+C	No. of CDS	AMR gene(s)^*a*^	Other resistance gene(s)	Biofilm-relevant operon(s)^*b*^	Plasmid replicon(s)^*c*^	Plasmid maintenance	Accession no.
FAM21845 chromosome	4,901,989	51.0	4,812	β-Lactam resistance: *ampC* (*ampH*)	Arsenic resistance: *arsRBC*, *arsRDABC*	Cellulose synthesis: *bcsABZC*, *bcsEFG*			CP017220
				Other: RND efflux pumps	Silver resistance: *silRSE*, *silCFBAP*	Curli synthesis: *csgDEFG*, *csgBAC*			
					Copper resistance: *cusCFBA*, *cusRS*, *pcoABCDRSE*	PGA synthesis: *pgaABCD*			
					Other: see Table S3 in the supplemental material	Colanic acid synthesis: *wza* to *wcaL*			
pFAM21845_1	147,225	51.7	174	Tetracycline resistance: *tet*(*B*)	Mercury resistance: *merR*, *merTPCADE*		IncFIA	*pemIK*, *ccdAB*, *vapBC*, *hok/sok*, *srnB/C*	CP017221
				Aminoglycoside resistance: *strA* (2×), *strB* (2×), *aph*(*3*′)-*Ic*, *aph*(*4*)-*Ia*, *aac*(*3*)-*IVa*, *aadA1*	Disinfectant resistance: *qacE*Δ*1*		IncFIB (AP001918)		
				Trimethoprim resistance: *dfrA1*			IncFII (pCoo)		
				Sulfonamide resistance: *sul1*					
				β-Lactam resistance: *bla*_TEM-1_					
pFAM21845_2	54,159	44.9	57	β-Lactam resistance: *bla*_TEM-1_		Type III fimbriae: *mrkABCDF*	IncX1	*stbDE*	CP017222
pFAM21845_3	5,828	46.6	8						CP017223
pFAM21845_4	2,454	48.8	4						CP017224
pFAM21845_5	1,934	51.4	2						CP017225

a Antimicrobial resistance (AMR) genes were detected by use of the NCBI annotation pipeline, using BLAST searches against β-lactamase sequences (http://www.lahey.org/studies/; accessed October 2016), the ARG-ANNOT database (http://www.mediterranee-infection.com/article.php?laref=282&titer=arg-annot) (94), and ResFinder 2.1 with default settings (%ID threshold of 90.00% and minimum length of 60%) (https://cge.cbs.dtu.dk/services/ResFinder/; accessed October 2016) (91). *ampC* encodes a class C β-lactamase; *ampH* encodes a d-alanyl-d-alanine-carboxypeptidase/endopeptidase closely related to AmpC β-lactamases, with very-low-level β-lactamase activity (98). RND efflux pumps, resistance-nodulation-cell division efflux pumps (AcrAB, AcrAD, AcrEF, MdtABC, and MdtEF, each with TolC and CusCFBA) with broad substrate ranges including antimicrobials (43).

b PGA, poly-beta-1,6-*N*-acetyl-d-glucosamine (poly-β-1,6-GlcNAc). The *bcs* operon lacks *bcsQ* and *bcsR*.

c Detected using PlasmidFinder 1.3 with default settings (95% ID threshold) (https://cge.cbs.dtu.dk/services/PlasmidFinder/; accessed October 2016) (92).

A total of five plasmids were sequenced and annotated. They are 147.2, 54.2, 5.8, 2.5, and 1.9 kb long and carry 174, 57, 8, 4, and 2 CDS, respectively. The first major plasmid is pFAM21845_1. It is a 147.2-kb conjugative IncFII plasmid (features all conjugal transfer *tra* and *trb* genes and *finO* of the IncFII reference plasmid pLV501 [all with E values of <10^−13^]). It carries antibiotic resistance genes against tetracyclines [*tet*(*B*)], aminoglycosides [*strA* (2×), *strB* (2×), *aph*(*3*′)-*Ic*, *aph* (*4*)-*Ia*, *aac*(*3*)-*IVa*, and *aadA1*], trimethoprim (*dfrA1*), sulfonamides (*sul1*), and β-lactams (*bla*_TEM-1_). The *mer* mercury resistance operon (*merR* and *merTPCADE* [48]) and the quaternary ammonium compound (QAC) resistance gene *qacEΔ1* are also located on this plasmid. We found no differences in the MIC of benzalkonium chloride between FAM21845, K-12 MG1655, and the pFAM21845_1 and pFAM21845_2 transconjugants (32 mg/liter for all strains). The 54.2-kb conjugative IncX1 plasmid pFAM21845_2 features a TEM-1 β-lactamase gene (*bla*_TEM-1_), and we found close homologs for all conjugative transfer proteins (apart from hypothetical ones) of the IncX reference plasmid R6K (all with E values of <10^−39^).

### FAM21845 locus of heat resistance.

**FIG 4:**
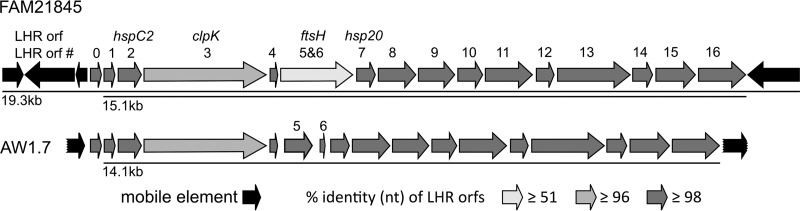
Comparison of loci of heat resistance of FAM21845 and AW1.7. The two loci are very similar overall (most ORFs have ≥98% identity at the nt level), with *clpK* having a slightly lower similarity (96.6%). A deletion in AW1.7 between *orf4* and *orf7* results in two ORFs where there is only one in FAM21845, carrying *ftsH*. Due to this 901-bp deletion, identity of the complete *ftsH* ORF at the nt level is only 51.9%. *orf5* and *orf6* of AW1.7, however, both map within the *ftsH* ORF of FAM21845, with high identity. The FAM21845 LHR features three mobile elements at the 5′ end (AW1.7 has only one), and both strains carry one 3′ mobile element. Note that the mobile elements of AW1.7 are partial ORFs on AW1.7 contig LDYJ01000141 (indicated by jagged ends).

The locus of heat resistance (LHR) is delineated by 5′ and 3′ mobile elements and carries a total of 16 open reading frames (ORFs), with a high degree of conservation. Of those with putative functions, we highlight *orf2*, encoding a small heat shock protein (HspC2); *orf3*, encoding the ATP-dependent Clp protease, ClpK, mentioned above; and *orf7*, encoding a heat shock protein, Hsp20 (12, 14). In this section, we compare the LHR found on the chromosome of FAM21845 to that of E. coli AW1.7 (accession no. LDYJ01000141). The two LHRs show a very high similarity overall (Fig. 4), even though the two strains were isolated from a raw milk cheese in Switzerland and a slaughter plant in Canada, respectively (49). They do differ in the region between *orf4* and *orf7*, where FAM21845 encodes the cell division protein FtsH (with the same length and only one amino acid change compared to the WP_000412529.1 sequence), while two separate putative ORFs (*orf5* and *orf6*) were detected in this region in AW1.7 (14) (Fig. 4). The identity of this region at the nucleotide level is only 51.9% due to a 901-bp deletion in AW1.7 compared to FAM21845. The deletion lies fully within the putative *ftsH* gene, and apart from it, the remaining 974 bp have only a single nucleotide mismatch between the two strains (99.9% identity). The LHR of FAM21845 is 15,080 bp long from the *orf1* to *orf16* homologs, with a GC content of 62.3% (that of AW1.7 is 14,087 bp long, with a GC content of 62.0%). Another difference between the two strains is the presence of three 5′ mobile elements in FAM21845 (as opposed to one in AW1.7). Both strains feature one 3′ mobile element. We found an additional ORF (*orf0*) in the same orientation as that of the entire LHR after the 5′ mobile elements before *orf1* in both strains. This 264-nucleotide (nt) ORF is almost identical between the two strains, with 95% (FAM21845) and 97% (AW1.7) identities on the amino acid level to a K. pneumoniae phospholipase (ALU57054.1). Including the three putative transposase genes and *orf0* at the 5′ end and the transposase gene at the 3′ end, the LHR of FAM21845 is 19,347 bp long, with a GC content of 60.7%. Although this GC content is lower than that for the narrower definition of the LHR (*orf1* to *orf16* only), it is still significantly above the 51.0% GC content of the chromosome overall (as previously noted for other E. coli LHRs) (14).

### FAM21845 genes involved in biofilm formation.

We used BLAST to compare all CDS of FAM21845 and its plasmids with a list of common biofilm-associated genes of E. coli (38). We found ORFs with high similarity for all genes (exclusively on the FAM21845 chromosome) except *ariR* (*ymgB*). *flu*, encoding the phase-variable biofilm formation autotransporter antigen 43 (Ag43), also yielded no hits with the strict criteria applied, as the longest high-scoring segment pair was only 44.2% of the query sequence BHT24_11475 (1,257 of 2,847 nt). More detailed investigation revealed that this high-scoring segment pair was so short due to six deletions in the putative Flu protein of FAM21845 (Flu_FAM21845_; 948 amino acids [aa]) compared to that of K-12 MG1655 (Flu_K-12_; 1,039 aa). The largest deletion is a stretch of 59 aa (positions 548 to 606 of Flu_K-12_). Overall sequence identity in a pairwise comparison was 74.4%. A Pfam domain search revealed very high similarity. Both proteins feature an N-terminal ESPR domain (extended signal peptide of type V secretion system; PF13018.3) and a C-terminal autotransporter β-domain (PF03797.16). Flu_FAM21845_ features four and Flu_K-12_ three AIDA domains (adhesin of bacterial autotransporter system, probable stalk; PF16168.2). FAM21845 carries an incomplete bacterial cellulose synthase (*bcs*) operon, featuring *bcsABZC* and *bcsEFG* but missing the NTPase gene *bcsQ* and the putative regulatory subunit gene *bcsR* (50). All genes of the operon required for curli synthesis (curli-specific genes [*csg*]), organized into *csgDEFG* and *csgBAC* (34, 51), were found in FAM21845. A direct comparison between the operons of FAM21845 and K-12 MG1655 revealed two major differences: (i) CsgD of FAM21845 is 203 aa long, while that of K-12 MG1655 is 216 aa long (there is a 13-aa deletion in FAM21845 at positions 2 to 14 of CsgD_K-12_; otherwise the proteins are 100% identical); and (ii) FAM21845 encodes the insertion element IS*1* protein InsB (identical to WP_001119291.1) in the intergenic region between *csgD* and *csgB*. The entire *csg* operon (from *csgG* to *csgC*) of FAM21845 is thus 4,733 nt long (4,443 nt in K-12 MG1655). Poly-β-1,6-*N*-acetyl-d-glucosamine (PGA) is another major constituent of the E. coli extracellular matrix that is involved in binding to abiotic surfaces and adhesion between cells (52). FAM21845 carries the full operon, *pgaABCD*. All 19 genes of the colanic acid synthesis operon (*wza* to *wcaL*), a further constituent of the E. coli extracellular matrix (53), are present in FAM21845 as well. We also screened all FAM21845 sequences for the presence of further fimbrial adhesion genes relevant to biofilms (54). Of the genes not previously discussed (i.e., those other than *csgA* and *csgD*), we found *fimA*, *fimE*, *elfA*, and *hcpA* (*ppdD*) on the chromosome, while no homologs of the E. coli common pilus gene *ecpA* were found. The only biofilm-related operon we found that is not located on the chromosome is the *mrkABCDF* operon, which encodes type III fimbriae (55).

### pFAM21845_2 increases biofilm formation in K-12 MG1655 transconjugants.

As both pFAM21845_1 and pFAM21845_2 are expected to be conjugative plasmids based on *in silico* data, we investigated their transfer in plate matings. Both plasmids were transferred to E. coli MG1655, but at statistically significantly different rates of 7.96 × 10^−5^ ± 5.55 × 10^−5^ and 2.84 × 10^−2^ ± 6.26 × 10^−3^ transconjugant per recipient for pFAM21845_1 and pFAM21845_2, respectively (*P* = 0.024; two-tailed, paired *t* test). As pFAM21845_1 encodes both ampicillin (AMP) and tetracycline (TET) resistances, we restreaked LB_NAL,RIF,AMP_-selected transconjugants (putatively carrying pFAM21845_2 only) onto LB_NAL,RIF,AMP,TET_ plates to assess possible cotransfer of pFAM21845_1. None of 120 restreaked colonies grew, demonstrating that the vast majority of transconjugants selected in this way are indeed positive only for pFAM21845_2, which is in line with the much higher transfer rate observed for this smaller plasmid. Transconjugants selected on LB_NAL,RIF,AMP,TET_ were sure to have received pFAM21845_1. Since transfer of this larger plasmid was approximately 3.6 × 10^2^ times less frequent than that of pFAM21845_2, we tested 49 of these transconjugants for the presence of both plasmids by specific PCRs. All 49 were positive by pFAM21845_1-specific PCR (as expected), while 20 were also positive for pFAM21845_2. Transfer of both plasmids, either separate or together, was thus observed. Both pFAM21845_1 and pFAM21845_2 feature plasmid maintenance systems and are therefore expected to be stably maintained (Table 3). As pFAM21845_2 features the *mrk* locus, which is known to increase biofilm formation (55), we performed CV assays on PS with wild-type K-12 MG1655 and its transconjugants to confirm this phenotype. Transfer of pFAM21845_2, but not pFAM21845_1, resulted in statistically significantly increased biofilm formation of the K-12 MG1655 transconjugant in 48-h CV assays on PS with ABTCAA at 12, 28, and 37°C (Table 4).

**TABLE 4:** Biofilm formation of K-12 MG1655 and its pFAM21845 transconjugants in ABTCAA on PS surfaces

Strain	12°C		28°C		37°C	
	OD_600_^*a*^	*P* value^*b*^	OD_600_^*a*^	*P* value^*b*^	OD_600_^*a*^	*P* value^*b*^
Wild-type K-12 MG1655	0.261		0.828		0.220	
K-12 MG1655(pFAM21845_1)	0.171	0.028	0.844	0.931	0.290	0.211
K-12 MG1655(pFAM21845_2)	1.955	<0.001	2.795	<0.001	1.678	<0.001

a Average OD_600_ of the indicated strain after subtraction of the OD_600_ of the negative control.

b Determined by one-way ANOVA, with wild-type K-12 MG1655 as a control (Holm-Sidak test).

## DISCUSSION

In this study, we analyzed 36 E. coli dairy isolates for their biofilm formation potential. We found very strong strain-specific differences with regard to all aspects of biofilm formation tested, even though all strains were isolated from raw milk cheeses (except for FAM22321 and FAM22871, which were isolated from raw milk). Curli and cellulose production levels in macrocolonies, for instance, ranged from no production under any conditions (FAM22936) to production of both under all conditions tested apart from RPSM_dil_ agar (FAM21843) (Fig. 1; see Table S1 in the supplemental material). We found fewer strains switching off curli and cellulose production in LBnoS than in LB when we increased the incubation temperature from 28 to 37°C (5 and 11 clear cases, respectively). This is consistent with both a temperature below 30°C and the absence of salt increasing *csgD* transcription and resulting in greater production of these two matrix constituents (37). A (largely) temperature-independent production of cellulose and curli (as is the case for FAM21843) has been observed previously (35). The overall biofilm formation scores of individual strains in CV assays on PS over all media and temperatures ranged from 4 (FAM22996) to 42 (FAM21845). We found ABTCAA to be the best medium in terms of maximizing biofilm formation, which is in agreement with previous observations (56). For each medium except RPSM_dil_, 28°C led to the greatest overall score for biofilm formation in CV assays. This observation makes sense, as E. coli strains capable of producing curli and cellulose normally do so more strongly at temperatures below 30°C (35). With 12°C being apparently too low to produce the same amount of biofilm within 48 h, it appears that 28°C is a good compromise between these two effects, generally leading to the most biofilm formation. In RPSM_dil_, part of the retained CV may be due to precipitated milk proteins caused by bacterial growth rather than to actual biofilm matrix or cells, as CV binds to proteins (57) as well as to DNA, peptidoglycan, and lipopolysaccharides (58). This may explain why 37°C was the best overall temperature with RPSM_dil_ in 96-well PS plates and why every strain produced at least some biofilm in this medium, as determined by CV assays, even though there was no visible production of either curli or cellulose on RPSM_dil_ agar for any strain (Fig. S1).

While production of biofilm along the sides of Bioflux FC was well reproduced, its formation within the FC channel was inconsistent. For this reason, we had to categorize strains into those either consistently or inconsistently producing biofilm within the FC channel. The absolute area coverage percentage varied greatly between replicates, even for strains which consistently produced biofilm within the FC channel. Some examples to illustrate this point are as follows. The average area coverage of FAM22936 peaked starting at 13.5 h (19.9%), reaching a maximum of 29.7% at 15 h, after which it decreased to 18.3% at 17 h (Fig. 3D). This peak was due mainly to one single replicate that peaked beginning at 13.5 h (28.8%), reached the maximum of 85.5% at 15 h, and then plummeted to 5.4% at 17 h. The other replicates of this strain were much more uniform over this period, with one having coverage ranging from 42.2 to 52.5% and the others having coverage of 30% and below, without pronounced peaks. FAM21843 exhibited a similarly extreme peak (22.6% at 18 h, increasing to 67.2% at 18.5 h, and decreasing back to 11.7% at 19 h), and another replicate showed a gradual increase to 49.1% at 18 h, with a drop to 11.2% at 18.5 h (Fig. 3B). FAM21805 averaged 44.5% area coverage after 24 h (Fig. 3C). This average is the result for replicates with area coverages as diverse as 92.9% and 5.4% at the 24-h time point. The second, lower replicate mentioned had gradually peaked earlier in the run, clearly having formed biofilm at that time (5.0% at 4 h, 26.9% at 8 h, and 10.3% at 13 h). The sudden drops in area coverage observed for several strains were likely the result of sloughing events, which are one mechanism of cell dispersion during the late stages of biofilm formation (59). As they did not occur in every replicate, and occurred at different times, they are certainly a contributor to the poor quantitative reproducibility of biofilm formation in FC (even for consistent biofilm formers). Even though K-12 MG1655 was not among the strongest biofilm formers under static conditions (overall CV score, 24) and showed statistically significantly lower IAR than those of both FAM21845 and FAM21805 (Fig. 2), it was clearly the strongest biofilm former in FC channels (Fig. 3C). It is interesting that 5 of the 11 consistent FC biofilm formers (FAM22936, FAM23012, FAM23092, FAM23093, and FAM23101) did not produce curli or cellulose under any condition tested.

In CV assays on SSC in full-strength RPSM, we observed significant staining of negative-control coupons. In this case, milk protein adherent to the steel surface was likely stained, even in the absence of any biofilm matrix or bacterial cells. However, strain FAM21845 stood out in that it was the only isolate we tested that exceeded this background level of CV staining enough to reach an average OD/ODc ratio of >1. Due to its interesting traits (highest biofilm formation on PS and SSC, highest IAR, and heat resistance and MDR phenotypes), FAM21845 was fully sequenced.

The complete genome sequence of FAM21845 revealed the presence of both the curli (*csg*) and cellulose (*bcs*) operons, which seem to be inactive (as judged by macrocolony assays). This cannot be due to nonfunctional RpoS (which is required for the transcription of these two operons [33]), as we indirectly ascertained its functionality in all E. coli strains used in this study via activity of catalase (30, 31). It is important that RpoS, depending on the exact background, can have positive (29) or negative (60) effects on biofilm formation. CsgD is required for transcription of *csgBAC*, and thus for curli synthesis, and also positively affects transcription of the *bcs* operon, and thus cellulose synthesis (61). The 13-aa deletion at the N terminus of CsgD_FAM21845_ and the insertion of a gene encoding the IS*1* protein InsB (100% identical to WP_001119291.1) between *csgD* and *csgB* likely negatively affect CsgD-mediated regulation. In addition, the *bcs* operon of FAM21845 lacks the NTPase gene *bcsQ* and the putative regulatory subunit gene *bcsR* (50). The possibly negatively affected CsgD-mediated regulation and incomplete *bcs* operon likely account for the observed curli- and cellulose-negative phenotype. In a 2005 study, curli-negative mutants clearly produced less biofilm and were unable to produce three-dimensional structures of a mature biofilm (62). FAM21845 does feature many other biofilm-related genes, however. The *pga* operon synthesizing PGA, involved in binding to abiotic surfaces and adhesion between cells (52), and the complete colanic acid synthesis operon (*wza* to *wcaL*) (53) are present on the chromosome. It is important that colanic acid can negatively affect biofilm formation by masking adhesins, such as Ag43 and AidA (63). In addition, FAM21845 encodes type III fimbriae (*mrk* operon) on pFAM21845_2, which increased biofilm formation in K-12 MG1655 transconjugants (Table 4). This was expected, as these fimbriae are known to increase biofilm formation (55), which we previously investigated for pFAM21805 as well (25). The benzalkonium chloride MIC of the pFAM21845_1 transconjugant (featuring *qacEΔ1*) was not increased, though. It was previously noted that the presence of *qacEΔ1* does not necessarily correlate with increased phenotypic QAC resistance (64), and this seems to be the case here as well. As both pFAM21845_1 and pFAM21845_2 are conjugative plasmids, the strain can produce conjugative pili (experimentally proven by HGT experiments), which are associated with increased biofilm formation (65–67). Reisner and colleagues also showed that expression of the F conjugative pilus could functionally substitute for other known adhesion factors, such as type I pili, Ag43 (shortened version in FAM21845), or curli (66). Another study showed that even an engineered reduced-genome E. coli strain lacking curli, type I fimbriae, exopolysaccharide polymers, and the autoinducer-2 signaling molecule can produce mature biofilm (68). Taking these findings into account, it is not surprising that FAM21845 can be a strong biofilm former, even without curli and cellulose production.

The different biofilm formation assays correlated well for only some strains, and here we give some examples of good and poor correlations between the different assays. In addition to FAM21845, FAM23016 is another example of a strong biofilm former under static conditions (overall CV score, 33) which does not produce curli or cellulose and forms no biofilm within FC channels. In contrast, FAM23101 produces as much biofilm under static conditions as FAM23016 does and produces no curli or cellulose, but it consistently forms biofilm within FC channels. A consistent former of biofilm in FC channels does not necessarily have to be strong under static conditions, either, as exemplified by FAM22962, which had an overall CV score of 11 (rank of 34). The different biofilm formation assays correlated well for FAM22996, which is the weakest biofilm former in CV assays (overall score, 4), produces no curli or cellulose, and also forms no biofilm in FC channels. A good correlation between assays was also seen for FAM21843 (CV score, 30). This strain is among the stronger biofilm formers under static conditions (rank of 9), especially at 28°C and in LB, LBnoS, and ABTCAA (ranks of 1, 2, and 1, respectively for absolute OD/ODc ratios). It produces curli and cellulose on LB, LBnoS, and ABTCAA agar at both 28 and 37°C, and it also consistently forms biofilm in FC channels, even though its IAR is among the lowest we determined (Fig. 2).

This highly strain-specific behavior is plausible in light of E. coli being a very diverse species, with a core genome of only ∼1,700 genes (genome size of ∼4,800 genes) and a pan-genome of more than 16,000 gene clusters (69). Also, strong phenotypic differences in biofilm formation between E. coli strains are commonly observed (56, 70, 71). A lack of correlation between curli/cellulose production and biofilm formation (71, 72) and between cell hydrophobicity and resulting surface attachment behavior (73) has also been described. This helps to explain the imperfect correlation between macrocolony assays and biofilm formation. We found a consistent correlation only between cellulose production and CV score at 28°C (for all media except RPSM_dil_) (Table S2). Another source of the large differences observed between assays in this study may have been the different materials used: PS and SSC were used for biofilm formation under static conditions, PVC was used for IAR assays, and the FC channels used for biofilm formation under dynamic conditions were made from polydimethylsiloxane (PDMS) (sides and top) and glass (bottom). Note that inconsistent biofilm formation between static and dynamic conditions has been observed before (66). Expanding the Bioflux FC assays to include other media and temperatures, or the use of another FC system entirely, would likely yield different results under dynamic conditions, which may correlate better with the static assays in some cases.

We can conclude that the strain collective tested in this study is extremely diverse with regard to biofilm formation and that no single assay can adequately predict any given strain's behavior in another. Biofilm formation must therefore be assessed on a strain- and assay-specific basis, using very specific conditions, as noted before (74). We found that many E. coli isolates tested in this study are strong biofilm formers, with transferrable biofilm-relevant genes (*mrk* operon) in the case of FAM21845. This particular strain additionally harbors resistance genes against heat stress (LHR), disinfectants, antibiotics, and heavy metals, resulting in ample opportunity for coselection. This can occur by means of coresistance (resistance factors located on the same genetic element) or cross-resistance (one mechanism providing resistance against more than one agent) (75). Coresistance in FAM21845 occurs on both the chromosome (*ampC* and the *ars* and *sil* operons) and pFAM21845_1 [*tet*(*B*), *strA*, *dfrA1*, *bla*_TEM-1_, *mer* operon, and others] (Table 3). Cross-resistance in this strain is possibly due to resistance-nodulation-cell division (RND) efflux pumps (AcrAB, AcrAD, AcrEF, MdtABC, and MdtEF, each in combination with TolC and CusCFBA), all of which are present in FAM21845 and are known to have several substrates each (43). We note that copper vats, rather than stainless steel ones, are used in many traditional Swiss dairies and may result in some degree of selection pressure and increased transcription of the *cus* or *pco* operons. The possibilities for co- and cross-resistances, combined with drastically increased heat resistance and strong biofilm formation, make strains such as FAM21845 a serious concern for the spread of resistance and persistence factors in the food industry, and conceivably to commensal or pathogenic bacteria in the gastrointestinal tract upon consumption of contaminated foods (27, 28).

## MATERIALS AND METHODS

### Media.

The following media were used throughout this study: Luria-Bertani Lennox broth (LB; 10 g/liter peptone, 5 g/liter yeast extract, 5 g/liter NaCl, pH 7.0), LBnoS (LB without addition of NaCl), tryptic soy broth (TSB; Oxoid, Pratteln, Switzerland), AB minimal medium with 0.5% Casamino Acids as a carbon source (ABTCAA) (56), and reconstituted powdered skim milk (RPSM), either full strength (10.5% [wt/vol]) or diluted (RPSM_dil_) (0.2% [wt/vol]). All media except TSB and full-strength RPSM were also used as agar plates (1.2% [wt/vol] agar). Overnight (ON) cultures were grown at 37°C in LB if not otherwise indicated.

### Strain characterization.

Because biofilm formation by E. coli is significantly dependent on the stationary-phase sigma factor RpoS (29), we tested the activity of catalase as an indirect method to confirm RpoS-mediated transcription (30, 31). Catalase activity of E. coli strains was assessed by addition of a 3% H_2_O_2_ solution to streaked colony material on a glass slide. Visible bubble formation indicated the presence of catalase (O_2_ production). The phylogenetic groups of strains were determined by quadruplex and group C- and E-specific PCRs (76), and the sequence type was determined by use of a 7-allele multilocus sequence typing scheme (MLST Database at UoW) (77). Heat resistance of strains was determined by *clpK* and *orfI* PCRs and was phenotypically confirmed (<1-log reduction in CFU after 30 min of incubation at 55°C) as previously described (18). Antimicrobial resistance profiles were determined according to CLSI guidelines (78). The following antimicrobials were tested: gentamicin (GEN), kanamycin (KAN), streptomycin (STR), chloramphenicol (CHL), tetracycline (TET), nalidixic acid (NAL), ciprofloxacin (CIP), trimethoprim (TMP), sulfamethoxazole-trimethoprim (19:1; SXT), ampicillin (AMP), cefoxitin (FOX), cephalothin (CEF), cefuroxime (CXM), cefotaxime (CTX), cefepime (FEP), aztreonam (ATM), amoxicillin-clavulanic acid (20:10; AMC), and ertapenem (ETP). All strains used in this study and their relevant characteristics are given in Table 1. Note that many of the strains have been part of previous studies (most recently, the studies detailed in reference 18), with a focus on heat resistance and AMR, but *rpoS* functionality, curli/cellulose production, and biofilm formation were not assessed.

### Crystal violet assays on polystyrene surfaces.

Biofilm formation was assessed by CV assays in 96-well plates (untreated PS surfaces) (CytoOne; StarLab, Hamburg, Germany). ON cultures of strains were diluted 1:100 in fresh medium, and 150 μl was added per well (eight wells per strain and biological replicate). Plates were incubated at 12, 28, and 37°C for 48 h. After incubation, plates were washed three times with 200 μl dilution solution (8 g/liter NaCl, 1 g/liter peptone) per well and subsequently stained with 200 μl 0.1% CV solution (Sigma-Aldrich, Buchs, Switzerland) per well for 20 min. Staining was followed by three washes with double-distilled water (ddH_2_O), and biofilms were dissolved in 200 μl 96% ethanol (EtOH) per well. Biofilm formation was assessed by measurement of the optical density at 600 nm (OD_600_). The assay was performed in biological triplicate. Biofilm formation results are reported in Table 2, using categories defined by OD/ODc ratios (79). Overall scores for media and strains are the sums of the category numbers in the columns and rows of the table, respectively. For comparison of wild-type K-12 MG1655 and its pFAM21845_1 and pFAM21845_2 transconjugants, triplicates of the values for OD_strain_ − OD_negative control_ were used (Table 4).

### Crystal violet assays on stainless steel coupons.

SSC (25 × 25 mm ± 1 mm; thickness, 1 mm ± 10%) (AISI 304 stainless steel; Goodfellow Cambridge Ltd., Huntingdon, England) were treated with professional cleaning-in-place products for 30 min at 55°C in an ultrasonic bath. Treatment with an alkaline cleaner (with a booster) was followed by acid cleaner treatment (Pasteurreiniger 405, Halaplus, and Halacid sauer, respectively; Halag Chemie AG, Aadorf, Switzerland). ON cultures of strains to be tested were diluted 1:100 in full-strength RPSM. Coupons were placed in 6-well plates (CytoOne; StarLab, Hamburg, Germany) and submerged in the inoculated medium. Plates were incubated for 48 h at 12°C, and the coupons were removed and washed three times by immersion and slight agitation in dilution solution. Coupons were stained by immersion in 0.1% CV solution (Sigma-Aldrich, Buchs, Switzerland) for 20 min and subsequently washed three times in ddH_2_O. The CV was removed by adding the coupons to 10 ml of modified biofilm-dissolving solution (MBDS; 10% SDS dissolved in 80% ethanol) (80) in a 50-ml tube and vortexing. Two 200-μl aliquots of the resulting stained MBDS were added to a 96-well microtiter plate for measurement of the OD_600_. The assay was done in technical duplicate (two coupons per strain) and biological triplicate, and results are reported as categories of OD/ODc ratios as for the assays done on PS surfaces in 96-well plates (Table 2).

### Determination of initial adhesion rates.

IAR on polyvinyl chloride (PVC) were determined essentially as previously described (81). A CoverWell perfusion chamber (19 × 6 × 0.5 mm; Invitrogen) was placed on top of a dry, uncoated PVC microscopy slide (treated in 70% EtOH-1% HCl ON and washed with ddH_2_O) and sealed with silicone lubricant. ON cultures (TSB, 37°C for 22 ± 2 h, 225 rpm) were diluted in citric acid-Na_2_HPO_4_ buffer (pH 6.6) to an OD_600_ of 0.100 ± 0.005 and pumped through the chamber at a pressure of 0.0505 Pa. Three separate vistas in the middle of the channel (along the *x* axis) were taken every 5 min for 30 min, using a 40× objective (Zeiss Fluar 40×/NA 1.3, oil immersion) and an inverted microscope with an automated stage (Zeiss Axiovert 135 TV). Cells were counted for each vista over time, and the median number of cells attached was determined for each time point. The initial adhesion rate was defined as the slope of the linear regression through the median number of attached cells over time [with a *y* intercept of 0 and units of cells per (minute × square centimeter)]. Measurements were done at least in biological duplicate.

### Biofilm assays under dynamic conditions.

Biofilm formation under flow conditions was assessed using the Fluxion Bioflux system (Fluxion Biosciences Inc., San Francisco, CA, USA). ON cultures of strains were centrifuged (2 min, 12,000 × *g*), and the supernatants were removed and resuspended in the same volume of ABTCAA minimal medium. We used 48-well low-shear plates (0 to 20 dynes/cm^2^). The cross sections of FC channels for biofilm formation are 350 μm wide and 70 μm tall. The channel roof and sides consist of PDMS, while the bottom is standard 180-μm coverslip glass. Channels were first wetted by adding 100 μl ABTCAA to the inlet well and applying force at 2 dynes/cm^2^ until small drops formed in the outlet well. Inoculation was done by adding 20 μl of resuspended cells to the outlet well and applying force at 2 dynes/cm^2^ for 2 s. Cells were allowed to adhere for 1 h at room temperature before 1 ml ABTCAA was added to each inlet well and the experiment was started. Runs were done at 37°C for 24 h by applying force at 0.15 dyne/cm^2^, with image acquisition every 30 min, using a 5× objective and a bright field. A technical duplicate (neighboring channels) and a minimum of three biological replicates were performed for each strain. The percent area coverage of the biofilm relative to the entire visible part of the channel (based on four to seven technical replicates) was determined for consistent biofilm formers by using Fiji (ImageJ 1.51g) (82).

### Macrocolony assays.

E. coli macrocolony assays were performed similarly to those in a previous study (83). Agar plates containing 40 μg/ml Congo red (Sigma-Aldrich, Buchs, Switzerland) and 20 μg/ml Coomassie brilliant blue G (Sigma-Aldrich, Buchs, Switzerland) or 0.01% calcofluor fluorescent brightener 28 (calcofluor; Sigma-Aldrich, Buchs, Switzerland) were spotted with 5 μl ON cultures of E. coli strains and incubated at 28 and 37°C for 7 and 3 days, respectively. LB, LBnoS, RPSM_dil_, and ABTCAA agars were used with both staining methods. LB and LBnoS were also used without addition of any dyes.

### Sequencing and assembly of the FAM21845 genome.

The genome of FAM21845 was sequenced with PacBio SMRT technology (one SMRT cell, P6-C4 chemistry, size selection with BluePippin to allow for long reads), resulting in a total of 1.47 Gbp of sequence data. After quality filtering, 53,900 reads with a mean subread length of 10,225 bp were obtained. Subsequent *de novo* genome assembly and resequencing steps were performed as described previously (84). Terminal repeats were removed, and the genome was circularized using Circlator 1.1.2 (85). Several rounds of sequence polishing using PacBio SMRT Portal protocol RS_Resequencing.1 were performed, resulting in one 4,901,989-bp chromosome and two large plasmids, with lengths of 147,225 bp and 54,159 bp (Table 3). The average coverage after the last resequencing step was 62-fold. To capture additional small plasmids and to do error correction on the initial PacBio assembly, a sequencing run using Illumina MiSeq was performed (paired-end sequencing; 2 × 300 bp). Raw reads were quality and adapter trimmed by employing Trimmomatic 0.32 (86). Trimmed reads were subsequently mapped to the initial PacBio assembly, and a subset of the unmapped reads was used for plasmid assembly using plasmidSPAdes 3.9.0 (87). Three additional small plasmids (5,828 bp, 2,454 bp, and 1,934 bp) resulted from the plasmidSPAdes assembly (Table 3). For error correction, the trimmed MiSeq reads were mapped to the chromosome and all five plasmids. Using freebayes 1.0.2-33 (88), eight single nucleotide indels were detected and corrected.

### Genome analysis of FAM21845.

The final FAM21845 genome and plasmid sequences were annotated by use of the NCBI pipeline (89) and further analyzed using CLC Genomics Workbench (v9.5; Qiagen, Hombrechtikon, Switzerland) and NCBI BLASTX (https://blast.ncbi.nlm.nih.gov/Blast.cgi) (90). The following tools for *in silico* analysis, hosted at the Center for Genomic Epidemiology (http://www.genomicepidemiology.org/), were used for further characterization: ResFinder 2.1 (with default settings of a %ID threshold of 90% and a minimum length of 60%; accessed October 2016) (91), PlasmidFinder 1.3 (with the default setting of a 95% ID threshold; accessed October 2016) (92), and MLST 1.8 (93), with MLST configuration Escherichia coli#1 (77). In cases where no online tool was used, BLASTx and tBLASTx analyses were carried out with the following strict criteria: identity of ≥90%, hit length of ≥90% of query, and E value of ≤10^−40^. These were used for detection of resistance genes (by use of the BacMet database [42; http://bacmet.biomedicine.gu.se]), biofilm formation-related genes (38), further resistance genes (by use of the ARG-ANNOT database [94; http://www.mediterranee-infection.com/article.php?laref=282&titer=arg-annot]), reference plasmids pLV501 (IncFII) and R6K (IncX) (http://www.sanger.ac.uk/resources/downloads/plasmids/), and all β-lactamases available at www.lahey.org/study with an accession number and were complemented by NCBI BLAST searches. Incomplete (resistance) operons detected with these strict criteria were manually curated with a lower percent ID threshold (>60%) to reduce the chance of false-negative results. Pfam domains of proteins were detected using CLC Genomics Workbench (database Pfam-A v29; predicted by HMMER 3.1b1 [May 2013]).

### Horizontal gene transfer experiments.

Horizontal gene transfer of FAM21845 plasmids carrying AMR genes was assessed by plate matings performed as previously described (18). An E. coli K-12 MG1655 NAL- and rifampin (RIF)-resistant mutant (95) was used as the recipient. Transconjugants were selected on LB supplemented with NAL, RIF, and AMP (LB_NAL,RIF,AMP_) or NAL, RIF, AMP, and TET (LB_NAL,RIF,AMP,TET_). Concentrations of antibiotics used were 30 μg/ml for NAL, 100 μg/ml for RIF, 100 μg/ml for AMP, and 15 μg/ml for TET. All antibiotics were purchased from Sigma-Aldrich (Buchs, Switzerland). Transconjugants were identified as K-12 by a PCR using primers K12-R (5′-ATCCTGCGCACCAATCAACAA-3′) and K12IS-L (5′-CGCGATGGAAGATGCTCTGTA-3′) (96). The two AMR plasmids of FAM21845 were detected using primers pFAM21845_1_ch_F (5′-TTTGGTGCACACGAGTATTGAGC-3′) plus pFAM21845_1_ch_R (5′-CCTTCCTTGGCGAGCATTGG-3′) and pFAM21845_2_ch_F (5′-CAAAAATACTTCTCCTTGCAGACG-3′) plus pFAM21845_2_ch_R (5′-TGATTTCTTCAGGTGTGATAGTCG-3′).

### Broth microdilution susceptibility testing.

FAM21845, K-12 MG1655, and the pFAM21845_1 and pFAM21845_2 transconjugants were tested for QAC susceptibility by use of benzalkonium chloride in 2-fold dilution steps (0.125 to 1,024 mg/liter) in cation-adjusted Mueller-Hinton broth according to the CLSI broth microdilution guidelines (97).

### Statistical analyses.

Statistical comparisons of data sets (*t* test, Mann-Whitney rank sum test, one-way ANOVA, and Spearman rank order correlation, as indicated in the text or legends) were performed using SigmaPlot 13.0 (Systat Software, San Jose, CA). The Spearman rank order test was performed on the data depicted in Table S1 in the supplemental material. CV score values were used directly. Results of “no” or “−” were replaced with “0,” those of “yes,” “+,” or “(+)” with “1,” and those of “yes/no” with “0.5.” FAM22947 was excluded from analysis in ABTCAA (both temperatures) because its curli production was uncertain.

### Figures.

Figures were created using Inkscape (v0.92; www.inkscape.org) and SigmaPlot 13.0.

### Accession number(s).

The sequences of the FAM21845 genome (1 chromosome and 5 plasmids) have been deposited in GenBank under accession no. CP017220 to CP017225.

## Supplementary Material

Supplemental material
