# Supplementary material for: Biofilm Formation Potential of Heat-Resistant Escherichia coli Dairy Isolates and the Complete Genome of Multidrug-Resistant, Heat-Resistant Strain FAM21845
Source: Appl Environ Microbiol. 2017 Jul 17;83(15):e00628-17. doi: 10.1128/AEM.00628-17 (PMC5514686; doi:10.1128/AEM.00628-17)
Supplement: Supplemental material [file supp_83_15_e00628-17__index.html]

Biofilm Formation Potential of Heat-Resistant Escherichia coli Dairy Isolates and the Complete Genome of Multidrug-Resistant, Heat-Resistant Strain FAM21845 — Supplemental material 

# Biofilm Formation Potential of Heat-Resistant Escherichia coli Dairy Isolates and the Complete Genome of Multidrug-Resistant, Heat-Resistant Strain FAM21845

## Supplemental material

- Supplemental file 1 -

  Comparison of curli and cellulose production, CV scores, and biofilm formation in flow cells of all strains tested in this study (Table S1), significant correlations by Spearman rank order test (Table S2), BacMet results for FAM21845 (Table S3), and macrocolony assays of all 37 *E. coli* strains tested in this study (Fig. S1).

  PDF, 5.4M
