# Supplementary material for: Biofilm Formation Potential of Heat-Resistant Escherichia coli Dairy Isolates and the Complete Genome of Multidrug-Resistant, Heat-Resistant Strain FAM21845
Source: Appl Environ Microbiol. 2017 Jul 17;83(15):e00628-17. doi: 10.1128/AEM.00628-17 (PMC5514686; doi:10.1128/AEM.00628-17)
Supplement: Supplemental material [file AEM.00628-17_zam999117964s1.pdf]

## Supplemental material

**TABLE S1** Comparison of curli and cellulose production, CV scores and biofilm formation in flow cells of all strains tested in this study

| Strain | Misc. | reduction in curli and/or cellulose production at 37 compared to 28°C | | | LB | | | | | | LBnoS | | | | | | ABTCAA | | | | | | | | overall CV score |
|---|---|---|---|---|---|---|---|---|---|---|---|---|---|---|---|---|---|---|---|---|---|---|---|---|---|
| | | | | | 28°C | | | 37°C | | | 28°C | | | 37°C | | | 28°C | | | 37°C | | | | | |
| | | LB | LBnoS | ABTCAA | curli | cellulose | CV score | curli | cellulose | CV score | curli | cellulose | CV score | curli | cellulose | CV score | curli | cellulose | CV score | curli | cellulose | CV score | BF in FC channel | FC reproducibility | |
| FAM19195 | heat resistant | (yes) | no | yes | + | + | 4 | + | + | - | + | + | 3 | + | + | - | + | + | 4 | + | - | 2 | no | good | 20 |
| FAM21805 | heat resistant | (yes) | no | no | + | - | 4 | + | - | 2 | + | - | 3 | + | - | 1 | - | - | 3 | - | - | 3 | yes[a] | good | 23 |
| FAM21807 | heat resistant | (yes) | no | no | + | - | 3 | + | - | - | + | - | 2 | + | - | 1 | - | - | 3 | - | - | 2 | no | good | 19 |
| FAM21808 | heat resistant | yes | (yes) | yes | + | - | - | - | - | - | + | - | - | + | - | - | + | - | 1 | - | - | 1 | yes / no | poor | 8 |
| FAM21843 | heat resistant | no | no | no | + | + | 4 | + | + | 2 | + | + | 4 | + | + | 1 | + | + | 4 | + | + | 3 | yes[a] | good | 30 |
| FAM21845 | heat resistant | no | no | no | - | - | 3 | - | - | 3 | - | - | 4 | - | - | 3 | - | - | 4 | - | - | 4 | no | good | 42 |
| FAM22636 | heat resistant | no | no | no | - | - | 3 | - | - | 1 | - | - | 2 | - | - | 1 | - | - | 2 | - | - | 3 | no | good | 19 |
| FAM22639 | heat resistant | no | no | no | - | - | 2 | - | - | 2 | - | - | 1 | - | - | 2 | - | - | 3 | - | - | 3 | no | good | 26 |
| FAM22791 | heat resistant | no | no | no | - | - | 2 | - | - | 2 | - | - | 2 | - | - | 2 | - | - | 2 | - | - | 2 | no | good | 23 |
| FAM22808 | heat resistant | yes | yes | yes | + | + | 4 | - | - | 1 | + | + | 4 | + | - | 1 | + | + | 3 | - | - | 2 | no | good | 24 |
| FAM22891 | heat resistant | no | yes | no | - | - | 2 | - | - | 1 | + | - | 2 | - | - | 1 | - | - | 2 | - | - | 4 | no | good | 27 |
| FAM22936 | heat resistant | no | no | no | - | - | 2 | - | - | 2 | - | - | 1 | - | - | 1 | - | - | 3 | - | - | 3 | yes[a] | good | 27 |
| FAM22940 | heat resistant | no | no | no | - | - | 1 | - | - | 1 | - | - | - | - | - | - | - | - | 2 | - | - | 2 | yes / no | poor | 13 |
| FAM22947 | heat resistant | (yes) | (yes) | no | + | - | 1 | + | - | 2 | + | - | 1 | + | - | 2 | ? | - | 3 | ? | - | 2 | no | good | 24 |
| FAM22954 | heat resistant | yes | no | yes | + | + | 4 | - | - | 2 | + | + | 4 | + | + | 2 | + | + | 4 | (+) | - | 4 | yes / no | poor | 34 |
| FAM22961 | heat resistant | no | no | no | - | - | 3 | - | - | 1 | - | - | 3 | - | - | 1 | - | - | 4 | - | - | 2 | yes / no | poor | 28 |
| FAM22962 | heat resistant | yes | no | yes | + | - | - | - | - | - | + | - | - | + | - | - | + | + | 4 | - | - | 2 | yes[a] | good | 11 |
| FAM22963 | heat resistant | no | no | no | - | - | 3 | - | - | 1 | - | - | 3 | - | - | 2 | - | - | 4 | - | - | 2 | no | good | 32 |
| FAM23012 | heat resistant | no | no | no | - | - | 3 | - | - | 3 | - | - | 2 | - | - | 2 | - | - | 3 | - | - | 4 | yes[a] | good | 31 |
| FAM23014 | heat resistant | no | no | no | - | - | 3 | - | - | 2 | - | - | 2 | - | - | 2 | - | - | 3 | - | - | 3 | no | good | 30 |
| FAM23016 | heat resistant | no | no | no | - | - | 3 | - | - | 2 | - | - | 4 | - | - | 2 | - | - | 4 | - | - | 3 | no | good | 33 |
| FAM23030 | heat resistant | no | no | no | - | - | 3 | - | - | 1 | - | - | 4 | - | - | 1 | - | - | 4 | - | - | 2 | no | good | 30 |
| FAM23031 | heat resistant | no | no | no | - | - | 1 | - | - | 1 | - | - | 1 | - | - | 1 | - | - | 3 | - | - | 1 | yes / no | poor | 16 |
| FAM23078 | heat resistant | yes | no | yes | + | - | 1 | - | - | 1 | + | - | 1 | + | - | - | + | - | 4 | - | - | 3 | no | poor | 15 |
| FAM23092 | heat resistant | no | no | no | - | - | 3 | - | - | 3 | - | - | 2 | - | - | 3 | - | - | 3 | - | - | 4 | yes[a] | good | 30 |
| FAM23093 | heat resistant | no | no | no | - | - | 3 | - | - | 2 | - | - | 3 | - | - | 2 | - | - | 3 | - | - | 3 | yes[a] | good | 28 |
| FAM23101 | heat resistant | no | no | no | - | - | 3 | - | - | 2 | - | - | 3 | - | - | 2 | - | - | 3 | - | - | 4 | yes[a] | good | 33 |
| FAM23106 | heat resistant | yes | no | yes | + | - | 1 | - | - | - | + | - | 1 | + | - | - | + | - | 4 | - | - | 3 | yes / no | poor | 14 |
| FAM23109 | heat resistant | yes | no | yes | + | + | 2 | - | - | 2 | + | + | 2 | + | + | 2 | + | + | 4 | - | - | 3 | no | good | 26 |
| FAM23113 | heat resistant | no | no | no | - | - | 3 | - | - | 2 | - | - | 2 | - | - | 2 | - | - | 3 | - | - | 3 | yes / no | poor | 30 |
| FAM21846 | heat sensitive | no | no | no | - | - | 1 | - | - | 1 | - | - | 3 | - | - | 1 | - | - | 3 | - | - | 3 | no | good | 15 |
| FAM22942 | heat sensitive | yes | yes | yes | + | (+) | 4 | - | - | 4 | + | + | 4 | + | - | 3 | + | - | 4 | - | - | 4 | yes[a] | good | 31 |
| FAM22956 | heat sensitive | yes | yes | yes | + | (+) | 4 | - | - | 4 | + | + | 3 | + | - | 4 | + | - | 3 | - | - | 3 | no | good | 31 |
| FAM22996 | heat sensitive | no | no | no | - | - | - | - | - | - | - | - | - | - | - | - | - | - | - | - | - | - | no | poor | 4 |
| FAM22321 | heat sensitive, ESBL | no | no | no | - | - | 1 | - | - | - | - | - | 1 | - | - | 1 | - | - | - | - | - | 1 | no | good | 10 |
| FAM22871 | heat sensitive, ESBL | yes | no | yes | + | + | 4 | + | - | 1 | + | + | 4 | + | + | 1 | + | + | 4 | (+) | - | 2 | yes[a] | good | 22 |
| K-12 MG1655 | heat ssensitive | yes | yes | yes | + | - | 4 | - | - | 1 | + | + | 4 | - | - | 1 | + | - | 4 | - | - | 2 | yes[a] | good | 24 |

[a] strains with consistent biofilm formation within the channel. Average area coverage graphs are given in **Fig. 3**

LB: Luria-Bertani Lennox (10 g/L peptone, 5 g/L yeast extract, 5 g/L NaCl, pH 7.0), LBnoS: LB without addition of NaCl, ABTCAA: AB minimal media with 0.5% casamino acids as carbon source, ESBL: extended-spectrum β-lactamase producer, CV score: category of biofilm formation on polystyrene in the given media and temperature combinations, overall CV score: sum of all CV scores of all media and temperatures tested (including 12°C and RPSMdil, as in Table 1), BF: biofilm, FC: flow cell

**TABLE S2** Significant correlations by Spearman rank order test between production of curli or cellulose, CV score and biofilm formation in flow cell channels for given media and temperature combinations

| Media | Temperature | Variable 1 | Variable 2 | Correlation coefficient | P-value |
|---|---|---|---|---|---|
| LB | 28°C | curli | cellulose | 0.602 | 0.0000883 |
| LB | 28°C | cellulose | CV score | 0.586 | 0.000154 |
| LB | 37°C | curli | cellulose | 0.543 | 0.000559 |
| LBnoS | 28°C | cellulose | heat resistance | -0.369 | 0.0246 |
| LBnoS | 28°C | curli | cellulose | 0.615 | 0.0000538 |
| LBnoS | 28°C | cellulose | CV score | 0.556 | 0.000392 |
| LBnoS | 37°C | curli | cellulose | 0.479 | 0.00289 |
| ABTCAA | 28°C | curli | cellulose | 0.653 | 0.0000128 |
| ABTCAA | 28°C | curli | CV score | 0.487 | 0.00275 |
| ABTCAA | 28°C | cellulose | CV score | 0.428 | 0.00951 |
| ABTCAA | 37°C | curli | cellulose | 0.478 | 0.00337 |

Spearman rank order test was performed on data depicted in Table S1. CV score values were used directly. ‘No’ / ‘-‘ were replaced with 0, ‘yes’ / ‘+’ / ‘(+)’ with 1 and ‘yes / no’ with 0.5. FAM22947 was excluded from analysis in ABTCAA (both temperatures), as its curli production was uncertain ‘(?)’.

**TABLE S3** BacMet results for FAM21845

| Location | Accession | Locus tag | Position | Description | Organism best hit in BacMet[a] | Gene |
|---|---|---|---|---|---|---|
| pFAM21845_1 | CP017221 | BHT24_24905 | 40568..40915 | quaternary ammonium compound (QAC) resistance protein | Pseudomonas aeruginosa | *qacEΔ1* |
| pFAM21845_1 | CP017221 | BHT24_24965 | complement(53223.. 53459) | mercury resistance protein | - | *merE* |
| pFAM21845_1 | CP017221 | BHT24_24970 | complement(53456.. 53818) | mercuric resistance transcriptional repressor protein MerD | Serratia marcescens | *merD* |
| pFAM21845_1 | CP017221 | BHT24_24975 | complement(53836..55530) | Mercuric resistance protein MerA | Pseudomonas stutzeri | *merA* |
| pFAM21845_1 | CP017221 | BHT24_24980 | complement(55582.. 56004) | mercury transport protein MerC | Pseudomonas stutzeri | *merC* |
| pFAM21845_1 | CP017221 | BHT24_24985 | complement(56040..56315) | Mercuric transport protein periplasmic component MerP | Serratia marcescens | *merP* |
| pFAM21845_1 | CP017221 | BHT24_24990 | complement(56329..56679) | Mercuric transport protein MerT | Pseudomonas sp. K-62 | *merT* |
| pFAM21845_1 | CP017221 | BHT24_24995 | 56751..57185 | Mercuric resistance operon regulatory protein MerR | Pseudomonas aeruginosa | *merR* |
| Chromosome | CP017220 | BHT24_00265 | complement(54648..55025) | Protein ApaG | Salmonella typhimurium (strain LT2 / SGSC1412 / ATCC 700720) | *apaG* |
| Chromosome | CP017220 | BHT24_00285 | complement(58174..60528) | LPS-assembly protein LptD | Escherichia coli (strain K12) | *lptD* |
| Chromosome | CP017220 | BHT24_00630 | 140520..142070 | Blue copper oxidase CueO | Escherichia coli (strain K12) | *cueO* |
| Chromosome | CP017220 | BHT24_00975 | 215023..215733 | Lipoprotein NlpE | Escherichia coli (strain K12) | *nlpE* |
| Chromosome | CP017220 | BHT24_01515 | 309428..310378 | Magnesium transport protein CorA | Escherichia coli (strain K12) | *corA* |
| Chromosome | CP017220 | BHT24_02875 | complement(564429..567578) | Multidrug efflux pump subunit AcrB | Escherichia coli (strain K12) | *acrB* |
| Chromosome | CP017220 | BHT24_02880 | complement(567601..568794) | Multidrug efflux pump subunit AcrA | Escherichia coli (strain K12) | *acrA* |
| Chromosome | CP017220 | BHT24_02885 | 568936..569583 | HTH-type transcriptional regulator AcrR | Escherichia coli (strain K12) | *acrR* |
| Chromosome | CP017220 | BHT24_03005 | 597168..597575 | HTH-type transcriptional regulator CueR | Escherichia coli (strain K12) | *cueR* |
| Chromosome | CP017220 | BHT24_03025 | 599758..600537 | UPF0014 inner membrane protein YbbM | Escherichia coli (strain K12) | *ybbM* |
| Chromosome | CP017220 | BHT24_03315 | complement(667226..668674) | Sensor kinase CusS | Escherichia coli (strain K12) | *cusS* |
| Chromosome | CP017220 | BHT24_03320 | complement(668664..669347) | Transcriptional regulatory protein CusR | Escherichia coli (strain K12) | *cusR* |
| Chromosome | CP017220 | BHT24_03325 | 669504..670877 | Cation efflux system protein CusC | Escherichia coli (strain K12) | *cusC* |
| Chromosome | CP017220 | BHT24_03330 | 671035..671367 | Cation efflux system protein CusF | Escherichia coli (strain K12) | *cusF* |
| Chromosome | CP017220 | BHT24_03335 | 671383..672606 | Cation efflux system protein CusB | Escherichia coli (strain K12) | *cusB* |
| Chromosome | CP017220 | BHT24_03340 | 672618..675761 | Cation efflux system protein CusA | Escherichia coli (strain K12) | *cusA* |
| Chromosome | CP017220 | BHT24_03770 | complement(763235..764773) | Apolipoprotein N-acyltransferase | Escherichia coli (strain K12) | *lnt* |

| Location | Accession | Locus tag | Position | Description | Organism best hit in BacMet[a] | Gene |
|---|---|---|---|---|---|---|
| Chromosome | CP017220 | BHT24_03775 | complement(764798..765676) | Magnesium and cobalt efflux protein CorC | Salmonella typhimurium (strain LT2 / SGSC1412 / ATCC 700720) | *corC* |
| Chromosome | CP017220 | BHT24_04270 | complement(854680..855621) | Zinc transporter ZitB | Escherichia coli (strain K12) | *zitB* |
| Chromosome | CP017220 | BHT24_04320 | complement(864654..865442) | Transcriptional regulator ModE | Escherichia coli (strain K12) | *modE* |
| Chromosome | CP017220 | BHT24_04330 | 865887..866660 | Molybdate-binding periplasmic protein ModA | Escherichia coli (strain K12) | *modA* |
| Chromosome | CP017220 | BHT24_04340 | 867352..868410 | Molybdenum import ATP-binding protein ModC | Escherichia coli (strain K12) | *modC* |
| Chromosome | CP017220 | BHT24_04615 | 924256..924723 | Transcriptional regulator MntR | Escherichia coli (strain K12) | *mntR* |
| Chromosome | CP017220 | BHT24_04740 | 954746..955978 | Multidrug transporter MdfA | Escherichia coli (strain K12) | *mdfA* |
| Chromosome | CP017220 | BHT24_04785 | 962257..962979 | Oxygen-insensitive NADPH nitroreductase NfsA | Escherichia coli (strain K12) | *nfsA* |
| Chromosome | CP017220 | BHT24_05910 | complement(1197236..1198462) | Multidrug resistance protein MdtG | Escherichia coli (strain K12) | *mdtG* |
| Chromosome | CP017220 | BHT24_06200 | complement(1251173..1251805) | HTH-type transcriptional repressor ComR | Escherichia coli (strain K12) | *comR* |
| Chromosome | CP017220 | BHT24_06205 | 1252046..1252303 | Multiple stress resistance protein BhsA | Escherichia coli (strain K12) | *bhsA* |
| Chromosome | CP017220 | BHT24_06300 | complement(1272748..1273419) | Response regulator in two-component regulatory system with PhoQ | Klebsiella pneumoniae subsp. pneumoniae NTUH-K2044 | *phoP* |
| Chromosome | CP017220 | BHT24_06470 | complement(1298466..1298996) | Disulfide bond formation protein B | Escherichia coli (strain K12) | *dsbB* |
| Chromosome | CP017220 | BHT24_07080 | complement(1420994..1421782) | Enoyl-[acyl-carrier-protein] reductase [NADH] FabI | Escherichia coli (strain K12) | *fabI* |
| Chromosome | CP017220 | BHT24_07655 | 1545259..1546263 | Tellurite resistance protein TehA | Escherichia coli (strain K12) | *tehA* |
| Chromosome | CP017220 | BHT24_07660 | 1546260..1546853 | Tellurite methyltransferase TehB | Escherichia coli (strain K12) | *tehB* |
| Chromosome | CP017220 | BHT24_07990 | complement(1617558..1619093) | Probable glutamate/gamma-aminobutyrate antiporter GadC | Escherichia coli (strain K12) | *gadC* |
| Chromosome | CP017220 | BHT24_07995 | complement(1619249..1620649) | Glutamate decarboxylase beta GadB | Escherichia coli (strain K12) | *gadB* |
| Chromosome | CP017220 | BHT24_08030 | complement(1631527..1632288) | HTH-type transcriptional regulator YdeO | Escherichia coli (strain K12) | *ydeO* |
| Chromosome | CP017220 | BHT24_08040 | complement(1632808..1635087) | putative oxidoreductase YdeP | Escherichia coli (strain K12) | *ydeP* |
| Chromosome | CP017220 | BHT24_08210 | 1673729..1674163 | Multiple antibiotic resistance protein MarR | Escherichia coli (strain K12) | *marR* |
| Chromosome | CP017220 | BHT24_08215 | 1674178..1674567 | Multiple antibiotic resistance protein MarA | Escherichia coli (strain K12) | *marA* |
| Chromosome | CP017220 | BHT24_08650 | 1746792..1747112 | Acid shock protein | Escherichia coli (strain K12) | *asr* |
| Chromosome | CP017220 | BHT24_08665 | complement(1748248..1748577) | Spermidine export protein MdtI | Escherichia coli (strain K12) | *mdtI* |
| Chromosome | CP017220 | BHT24_08670 | complement(1748564..1748929) | Spermidine export protein MdtJ | Shigella sonnei (strain Ss046) | *mdtJ* |
| Chromosome | CP017220 | BHT24_08970 | 1811832..1812413 | Superoxide dismutase [Fe] SodB | Escherichia coli (strain K12) | *sodB* |

| Location | Accession | Locus tag | Position | Description | Organism best hit in BacMet[a] | Gene |
|---|---|---|---|---|---|---|
| Chromosome | CP017220 | BHT24_09010 | 1819984..1821357 | Multidrug resistance protein MdtK | Escherichia coli (strain K12) | *mdtK* |
| Chromosome | CP017220 | BHT24_09870 | 1986642..1987214 | Probable manganese efflux pump MntP | Escherichia coli (strain K12) | *mntP* |
| Chromosome | CP017220 | BHT24_10075 | complement(2022609..2023556) | High-affinity zinc uptake system protein ZnuA | Escherichia coli (strain K12) | *znuA* |
| Chromosome | CP017220 | BHT24_10080 | 2023620..2024375 | Zinc import ATP-binding protein ZnuC | Escherichia coli (strain K12) | *znuC* |
| Chromosome | CP017220 | BHT24_10085 | 2024372..2025157 | High-affinity zinc uptake system membrane protein ZnuB | Escherichia coli (strain K12) | *znuB* |
| Chromosome | CP017220 | BHT24_10515 | complement(2096796..2097542) | Copper homeostasis protein CutC | Escherichia coli (strain K12) | *cutC* |
| Chromosome | CP017220 | BHT24_10820 | complement(2152316..2152648) | Multidrug transporter EmrE | Escherichia coli (strain K12) | *emrE* |
| Chromosome | CP017220 | BHT24_11015 | 2182404..2183054 | Metal-binding protein ZinT | Escherichia coli (strain K12) | *zinT* |
| Chromosome | CP017220 | BHT24_11880 | 2345146..2346393 | Multidrug resistance protein MdtA | Escherichia coli (strain K12) | *mdtA* |
| Chromosome | CP017220 | BHT24_11885 | 2346393..2349515 | Multidrug resistance protein MdtB | Escherichia coli (strain K12) | *mdtB* |
| Chromosome | CP017220 | BHT24_11890 | 2349516..2352593 | Multidrug resistance protein MdtC | Escherichia coli (strain K12) | *mdtC* |
| Chromosome | CP017220 | BHT24_11900 | 2354006..2355409 | Signal transduction histidine-protein kinase BaeS | Escherichia coli (strain K12) | *baeS* |
| Chromosome | CP017220 | BHT24_11905 | 2355406..2356128 | Transcriptional regulatory protein BaeR | Escherichia coli (strain K12) | *baeR* |
| Chromosome | CP017220 | BHT24_12010 | complement(2374758..2375030) | Transcriptional repressor RcnR | Escherichia coli (strain K12) | *rcnR* |
| Chromosome | CP017220 | BHT24_12015 | 2375151..2375975 | Nickel/cobalt efflux system RcnA | Escherichia coli (strain K12) | *rcnA* |
| Chromosome | CP017220 | BHT24_12020 | 2376194..2376532 | Nickel/cobalt homeostasis protein RcnB | Escherichia coli (strain K12) | *rcnB* |
| Chromosome | CP017220 | BHT24_12420 | complement(2467657..2468847) | Bicyclomycin resistance protein | Escherichia coli (strain K12) | *bcr* |
| Chromosome | CP017220 | BHT24_13335 | complement(2665317..2666480) | Multidrug resistance protein K | Escherichia coli (strain K12) | *emrK* |
| Chromosome | CP017220 | BHT24_13340 | 2666896..2667510 | Positive transcription regulator EvgA | Escherichia coli (strain K12) | *evgA* |
| Chromosome | CP017220 | BHT24_13345 | 2667725..2671108 | Sensor protein EvgS | Escherichia coli O157:H7 | *evgS* |
| Chromosome | CP017220 | BHT24_13450 | complement(2692599..2693837) | Divalent metal cation transporter MntH | Escherichia coli (strain K12) | *mntH* |
| Chromosome | CP017220 | BHT24_13790 | 2757786..2760899 | RND family aminoglycoside/multidrug efflux pump | Salmonella typhimurium (strain LT2 / SGSC1412 / ATCC 700720) | *acrD* |
| Chromosome | CP017220 | BHT24_14830 | 2964519..2965049 | Transcriptional repressor MprA | Escherichia coli (strain K12) | *mprA* |
| Chromosome | CP017220 | BHT24_14835 | 2965176..2966348 | Multidrug resistance protein A | Escherichia coli (strain K12) | *emrA* |
| Chromosome | CP017220 | BHT24_15125 | complement(3020310..3021302) | RNA polymerase sigma factor RpoS | Shigella flexneri | *rpoS* |
| Chromosome | CP017220 | BHT24_15815 | complement(3162637..3163062) | Arsenate reductase | Escherichia coli | *arsC* |

| Location | Accession | Locus tag | Position | Description | Organism best hit in BacMet[a] | Gene |
|---|---|---|---|---|---|---|
| Chromosome | CP017220 | BHT24_15820 | complement(3163075..3164364) | Arsenical pump membrane protein | Acidiphilium multivorum | *arsB* |
| Chromosome | CP017220 | BHT24_15825 | complement(3164411..3166162) | Arsenical pump-driving ATPase | Acidiphilium multivorum (strain DSM 11245 / JCM 8867 / AIU301) | *arsA* |
| Chromosome | CP017220 | BHT24_15830 | complement(3166180..3166542) | Arsenical resistance operon trans-acting repressor ArsD | Escherichia coli | *arsD* |
| Chromosome | CP017220 | BHT24_15835 | complement(3166590..3166943) | Arsenical resistance operon repressor | Escherichia coli | *arsR* |
| Chromosome | CP017220 | BHT24_15945 | complement(3193678..3194388) | Thiol:disulfide interchange protein DsbC | Escherichia coli (strain K12) | *dsbC* |
| Chromosome | CP017220 | BHT24_16585 | complement(3313667..3314059) | Protein YgiW | Escherichia coli (strain K12) | *ygiW* |
| Chromosome | CP017220 | BHT24_16650 | 3323628..3325109 | Outer membrane protein TolC | Escherichia coli (strain K12) | *tolC* |
| Chromosome | CP017220 | BHT24_16675 | 3328063..3328836 | Zinc transporter ZupT | Escherichia coli (strain K12) | *zupT* |
| Chromosome | CP017220 | BHT24_16835 | complement(3359903..3360667) | NADPH-dependent ferric-chelate reductase YqjH | Escherichia coli (strain K12) | *yqjH* |
| Chromosome | CP017220 | BHT24_17945 | 3589967..3591124 | Acriflavine resistance protein E | Escherichia coli (strain K12) | *acrE* |
| Chromosome | CP017220 | BHT24_17950 | 3591136..3594240 | Acriflavine resistance protein F | Escherichia coli (strain K12) | *acrF* |
| Chromosome | CP017220 | BHT24_18085 | complement(3614817..3615242) | HTH-type transcriptional regulator ZntR | Escherichia coli (strain K12) | *zntR* |
| Chromosome | CP017220 | BHT24_19015 | 3790308..3791882 | Nickel-binding periplasmic protein NikA | Escherichia coli (strain K12) | *nikA* |
| Chromosome | CP017220 | BHT24_19020 | 3791882..3792826 | Nickel transport system permease protein NikB | Escherichia coli (strain K12) | *nikB* |
| Chromosome | CP017220 | BHT24_19025 | 3792823..3793656 | Nickel transport system permease protein NikC | Escherichia coli (strain K12) | *nikC* |
| Chromosome | CP017220 | BHT24_19030 | 3793656..3794420 | Nickel import ATP-binding protein NikD | Escherichia coli (strain K12) | *nikD* |
| Chromosome | CP017220 | BHT24_19035 | 3794417..3795223 | Nickel import ATP-binding protein NikE | Escherichia coli (strain K12) | *nikE* |
| Chromosome | CP017220 | BHT24_19040 | 3795229..3795630 | Nickel-responsive regulator | Escherichia coli (strain K12) | *nikR* |
| Chromosome | CP017220 | BHT24_19115 | complement(3813197..3813631) | Probable copper-binding protein PcoE | Escherichia coli | *pcoE* |
| Chromosome | CP017220 | BHT24_19120 | complement(3813849..3815249) | Probable sensor protein PcoS | Escherichia coli | *pcoS* |
| Chromosome | CP017220 | BHT24_19125 | complement(3815246..3815926) | Transcriptional regulatory protein PcoR | Escherichia coli | *pcoR* |
| Chromosome | CP017220 | BHT24_19130 | complement(3815981..3816910) | Copper resistance protein D | Escherichia coli | *pcoD* |
| Chromosome | CP017220 | BHT24_19135 | complement(3816915..3817295) | Copper resistance protein C | Escherichia coli | *pcoC* |
| Chromosome | CP017220 | BHT24_19140 | complement(3817335..3818231) | Copper resistance protein B | Escherichia coli | *pcoB* |
| Chromosome | CP017220 | BHT24_19145 | complement(3818231..3820048) | Copper resistance protein A | Escherichia coli | *pcoA* |
| Chromosome | CP017220 | BHT24_19170 | complement(3822030..3824471) | Silver exporting P-type ATPase | Salmonella typhimurium | *silP* |

| Location | Accession | Locus tag | Position | Description | Organism best hit in BacMet[a] | Gene |
|---|---|---|---|---|---|---|
| Chromosome | CP017220 | BHT24_19175 | complement(3824599.. 3825039) | Hypothetical protein in *sil* operon | - | *Orf105* |
| Chromosome | CP017220 | BHT24_19180 | complement(3825126..3828272) | Putative cation efflux system protein SilA | Salmonella typhimurium | *silA* |
| Chromosome | CP017220 | BHT24_19185 | complement(3828283..3829575) | Putative membrane fusion protein SilB | Salmonella typhimurium | *silB* |
| Chromosome | CP017220 | BHT24_19190 | complement(3829689.. 3830042) | periplasmic silver-binding protein SilF | Escherichia coli NCTC 50110 | *silF* |
| Chromosome | CP017220 | BHT24_19195 | complement(3830071..3831456) | Probable outer membrane lipoprotein SilC | Salmonella typhimurium | *silC* |
| Chromosome | CP017220 | BHT24_19200 | 3831646..3832326 | Probable transcriptional regulatory protein SilR | Salmonella typhimurium | *silR* |
| Chromosome | CP017220 | BHT24_19205 | 3832319..3833800 | Probable sensor kinase SilS | Salmonella typhimurium | *silS* |
| Chromosome | CP017220 | BHT24_19210 | 3834045..3834476 | Silver-binding protein SilE | Salmonella typhimurium | *silE* |
| Chromosome | CP017220 | BHT24_19265 | 3846172..3847671 | Low-affinity inorganic phosphate transporter 1 PitA | Escherichia coli (strain K12) | *pitA* |
| Chromosome | CP017220 | BHT24_19315 | 3857059.. 3857412 | Arsenical resistance operon repressor | Escherichia coli (strain K12) | *arsR* |
| Chromosome | CP017220 | BHT24_19320 | 3857466..3858755 | Arsenical pump membrane protein | Escherichia coli | *arsB* |
| Chromosome | CP017220 | BHT24_19325 | 3858768..3859193 | Arsenate reductase | Acidiphilium multivorum (strain DSM 11245 / JCM 8867 / AIU301) | *arsC* |
| Chromosome | CP017220 | BHT24_19355 | complement(3863741..3864073) | Acid stress chaperone HdeA | Escherichia coli (strain K12) | *hdeA* |
| Chromosome | CP017220 | BHT24_19365 | 3865699..3866226 | Transcriptional regulator GadE | Escherichia coli (strain K12) | *gadE* |
| Chromosome | CP017220 | BHT24_19375 | 3866565..3867722 | Multidrug resistance protein MdtE | Escherichia coli (strain K12) | *mdtE* |
| Chromosome | CP017220 | BHT24_19380 | 3867747..3870860 | Multidrug resistance protein MdtF | Escherichia coli (strain K12) | *mdtF* |
| Chromosome | CP017220 | BHT24_19385 | complement(3871223..3871951) | HTH-type transcriptional regulator GadW | Escherichia coli (strain K12) | *gadW* |
| Chromosome | CP017220 | BHT24_19390 | complement(3872319..3873143) | HTH-type transcriptional regulator GadX | Escherichia coli (strain K12) | *gadX* |
| Chromosome | CP017220 | BHT24_19395 | complement(3873513..3874913) | Glutamate decarboxylase alpha GadA | Escherichia coli (strain K12) | *gadA* |
| Chromosome | CP017220 | BHT24_20240 | 4057095..4058279 | Multidrug resistance protein D | Escherichia coli (strain K12) | *emrD* |
| Chromosome | CP017220 | BHT24_20285 | complement(4067232..4067660) | Small heat shock protein IbpB | Escherichia coli (strain K12) | *ibpB* |
| Chromosome | CP017220 | BHT24_20290 | complement(4067772..4068185) | Small heat shock protein IbpA | Escherichia coli (strain K12) | *ibpA* |
| Chromosome | CP017220 | BHT24_20485 | complement(4105184..4105957) | Phosphate import ATP-binding protein PstB | Escherichia coli (strain K12) | *pstB* |
| Chromosome | CP017220 | BHT24_20490 | complement(4106140..4107030) | Phosphate transport system permease protein PstA | Escherichia coli (strain K12) | *pstA* |
| Chromosome | CP017220 | BHT24_20495 | complement(4107030..4107989) | Phosphate transport system permease protein PstC | Escherichia coli (strain K12) | *pstC* |
| Chromosome | CP017220 | BHT24_20500 | complement(4108076..4109116) | Phosphate-binding protein PstS | Escherichia coli (strain K12) | *pstS* |
| Chromosome | CP017220 | BHT24_20925 | 4196759..4197709 | Magnesium transport protein CorA | Escherichia coli (strain K12) | *corA* |

| Location | Accession | Locus tag | Position | Description | Organism best hit in BacMet[a] | Gene |
|---|---|---|---|---|---|---|
| Chromosome | CP017220 | BHT24_21135 | 4238364..4238990 | Thiol:disulfide interchange protein DsbA | Escherichia coli (strain K12) | *dsbA* |
| Chromosome | CP017220 | BHT24_21390 | 4290951..4291571 | Superoxide dismutase [Mn] SodA | Escherichia coli (strain K12) | *sodA* |
| Chromosome | CP017220 | BHT24_21410 | complement(4293743..4295116) | Sensor protein of stress-related two-component regulatory system CpxA | Klebsiella pneumoniae subsp. pneumoniae NTUH-K2044 | *cpxA* |
| Chromosome | CP017220 | BHT24_21415 | complement(4295113..4295811) | Response regulator of stress-related two-component regulatory system CpxR | Klebsiella pneumoniae subsp. pneumoniae NTUH-K2044 | *cpxR* |
| Chromosome | CP017220 | BHT24_21425 | 4296609..4297511 | Ferrous-iron efflux pump FieF | Escherichia coli (strain K12) | *fieF* |
| Chromosome | CP017220 | BHT24_21485 | complement(4307385..4308230) | Glycerol uptake facilitator protein GlpF | Escherichia coli (strain K12) | *glpF* |
| Chromosome | CP017220 | BHT24_21665 | 4350075..4350992 | Activator of hydrogen peroxide-inducible genes | Klebsiella pneumoniae subsp. pneumoniae NTUH-K2044 | *oxyR* |
| Chromosome | CP017220 | BHT24_21880 | 4393704..4395029 | Transcriptional regulatory protein ZraR | Escherichia coli (strain K12) | *zraR* |
| Chromosome | CP017220 | BHT24_21950 | complement(4412271..4413095) | Acetate operon repressor | Escherichia coli (strain K12) | *iclR* |
| Chromosome | CP017220 | BHT24_22110 | complement(4448967..4449482) | Zinc uptake regulation protein | Escherichia coli (strain K12) | *zur* |
| Chromosome | CP017220 | BHT24_22490 | complement(4505078..4505401) | Regulatory protein SoxS | Escherichia coli (strain K12) | *soxS* |
| Chromosome | CP017220 | BHT24_22495 | 4505487..4505951 | Redox-sensitive transcriptional activator SoxR | Escherichia coli (strain K12) | *soxR* |
| Chromosome | CP017220 | BHT24_22515 | complement(4511270..4512919) | Cation/acetate symporter ActP | Escherichia coli (strain K12) | *actP* |
| Chromosome | CP017220 | BHT24_22595 | complement(4530757..4531788) | Multidrug resistance protein MdtN | Escherichia coli (strain K12) | *mdtN* |
| Chromosome | CP017220 | BHT24_22880 | complement(4586782..4587120) | Divalent-cation tolerance protein CutA | Escherichia coli (strain K12) | *cutA* |
| Chromosome | CP017220 | BHT24_22945 | 4598635..4598952 | Quaternary ammonium compound-resistance protein SugE | Escherichia coli (strain K12) | *sugE* |
| Chromosome | CP017220 | BHT24_23645 | 4724436..4727132 | Magnesium-transporting ATPase, P-type 1 | Escherichia coli (strain K12) | *mgtA* |
| Chromosome | CP017220 | BHT24_23860 | complement(4764262..4765029) | Fe(3+) dicitrate transport ATP-binding protein FecE | Escherichia coli (strain K12) | *fecE* |
| Chromosome | CP017220 | BHT24_24095 | complement(4804501..4805733) | Multidrug resistance protein MdtM | Escherichia coli (strain K12) | *mdtM* |
| Chromosome | CP017220 | BHT24_24635 | complement(4894782..4895651) | Right origin-binding protein | Escherichia coli (strain K12) | *rob* |
| Chromosome | CP017220 | BHT24_14840 | 2966365..2967903 | Multidrug resistance protein B EmrB | Escherichia coli (strain K12) | *emrB* |
| Chromosome | CP017220 | BHT24_13330 | complement(2663779..2665317) | Multidrug resistance protein Y | Escherichia coli (strain K12) | *emrY* |
| Chromosome | CP017220 | BHT24_23865 | complement(4765030..4765986) | Fe(3+) dicitrate transport system permease protein FecD | Escherichia coli (strain K12) | *fecD* |
| Chromosome | CP017220 | BHT24_19350 | complement(3863298..3863624) | Acid stress chaperone HdeB | Escherichia coli (strain K12) | *hdeB* |

| Location | Accession | Locus tag | Position | Description | Organism best hit in BacMet[a] | Gene |
|---|---|---|---|---|---|---|
| Chromosome | CP017220 | BHT24_04335 | 866660..867349 | Molybdenum transport system permease protein ModB | Escherichia coli (strain K12) | *modB* |
| Chromosome | CP017220 | BHT24_18980 | 3783093..3785291 | Lead, cadmium, zinc and mercury-transporting ATPase | Escherichia coli (strain K12) | *zntA* |

[a] rows without an entry did not have hits with the very strict criteria and were manually curated using lower ID percentage threshold (>60%) to reduce the chance of false negatives (see Methods).

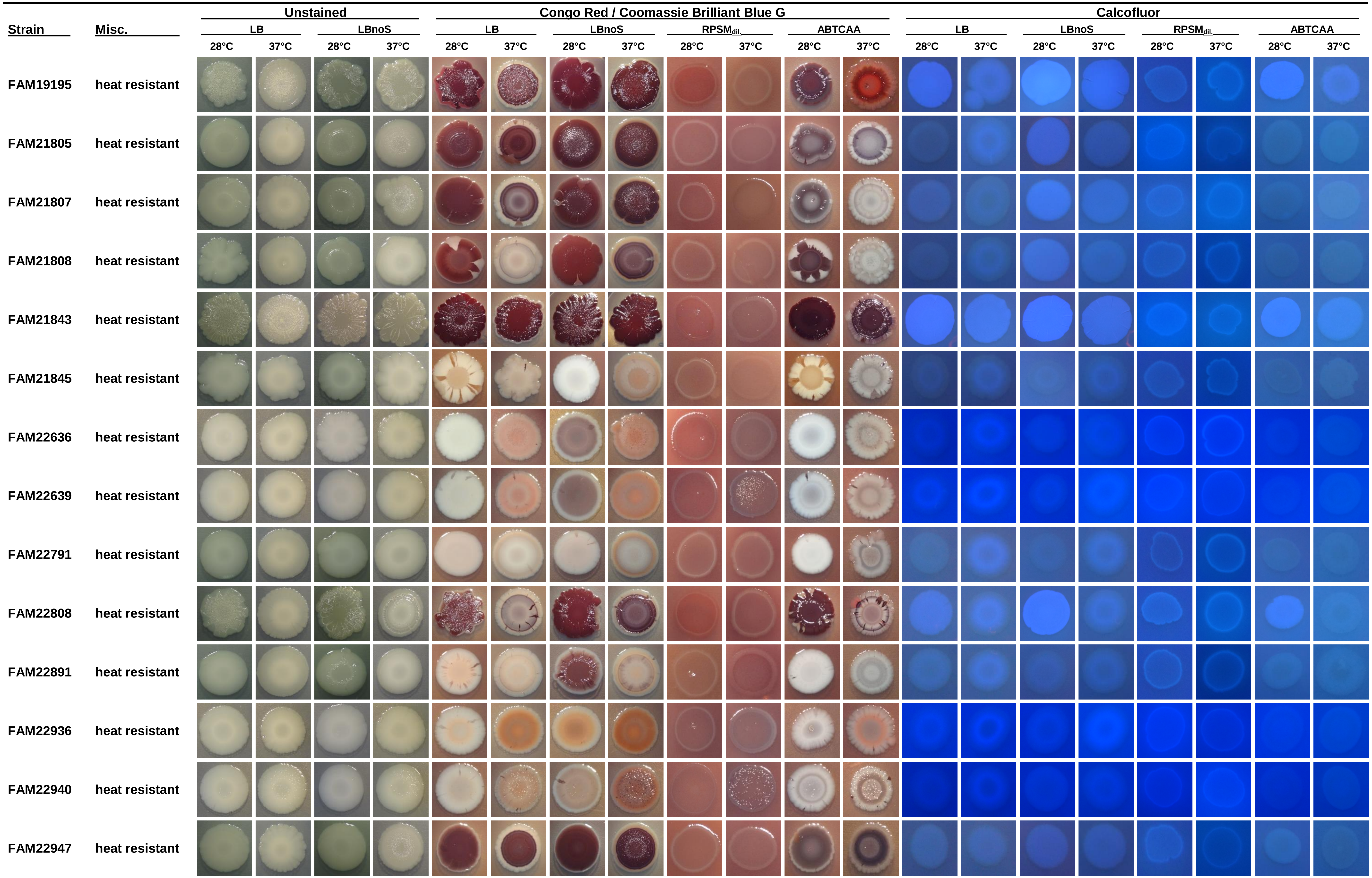

Strain
Misc.
Unstained
Congo Red / Coomassie Brilliant Blue G
Calcofluor
LB
LBnoS
LB
LBnoS
$RPSM_{dil.}$
ABTCAA
LB
LBnoS
$RPSM_{dil.}$
ABTCAA
28°C
37°C
FAM19195 heat resistant
FAM21805 heat resistant
FAM21807 heat resistant
FAM21808 heat resistant
FAM21843 heat resistant
FAM21845 heat resistant
FAM22636 heat resistant
FAM22639 heat resistant
FAM22791 heat resistant
FAM22808 heat resistant
FAM22891 heat resistant
FAM22936 heat resistant
FAM22940 heat resistant
FAM22947 heat resistant

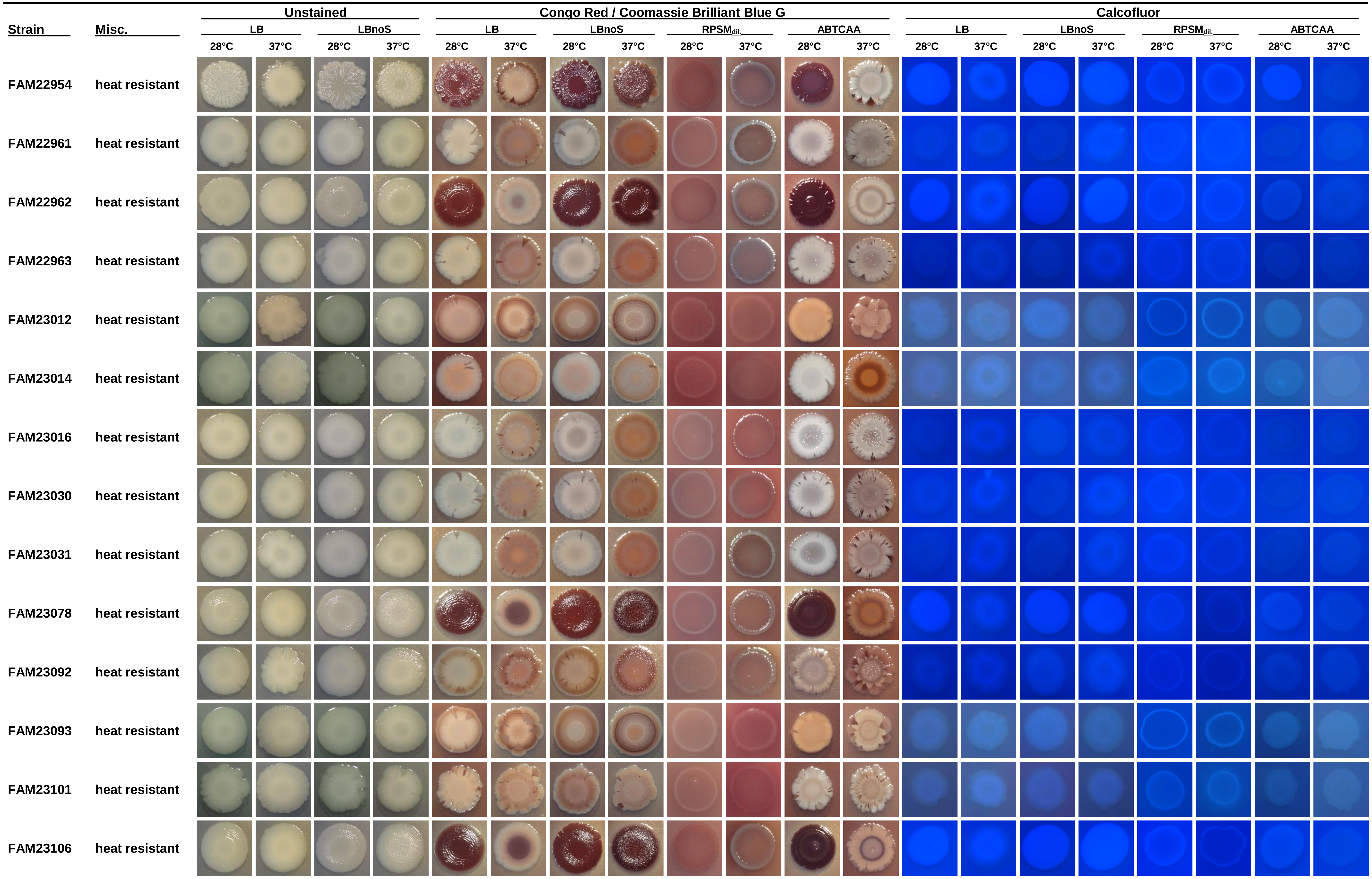
Strain
Misc.
Unstained
Congo Red / Coomassie Brilliant Blue G
Calcofluor
LB
LBnoS
RPSM$_{dil.}$
ABTCAA
28°C
37°C
FAM22954 heat resistant
FAM22961 heat resistant
FAM22962 heat resistant
FAM22963 heat resistant
FAM23012 heat resistant
FAM23014 heat resistant
FAM23016 heat resistant
FAM23030 heat resistant
FAM23031 heat resistant
FAM23078 heat resistant
FAM23092 heat resistant
FAM23093 heat resistant
FAM23101 heat resistant
FAM23106 heat resistant

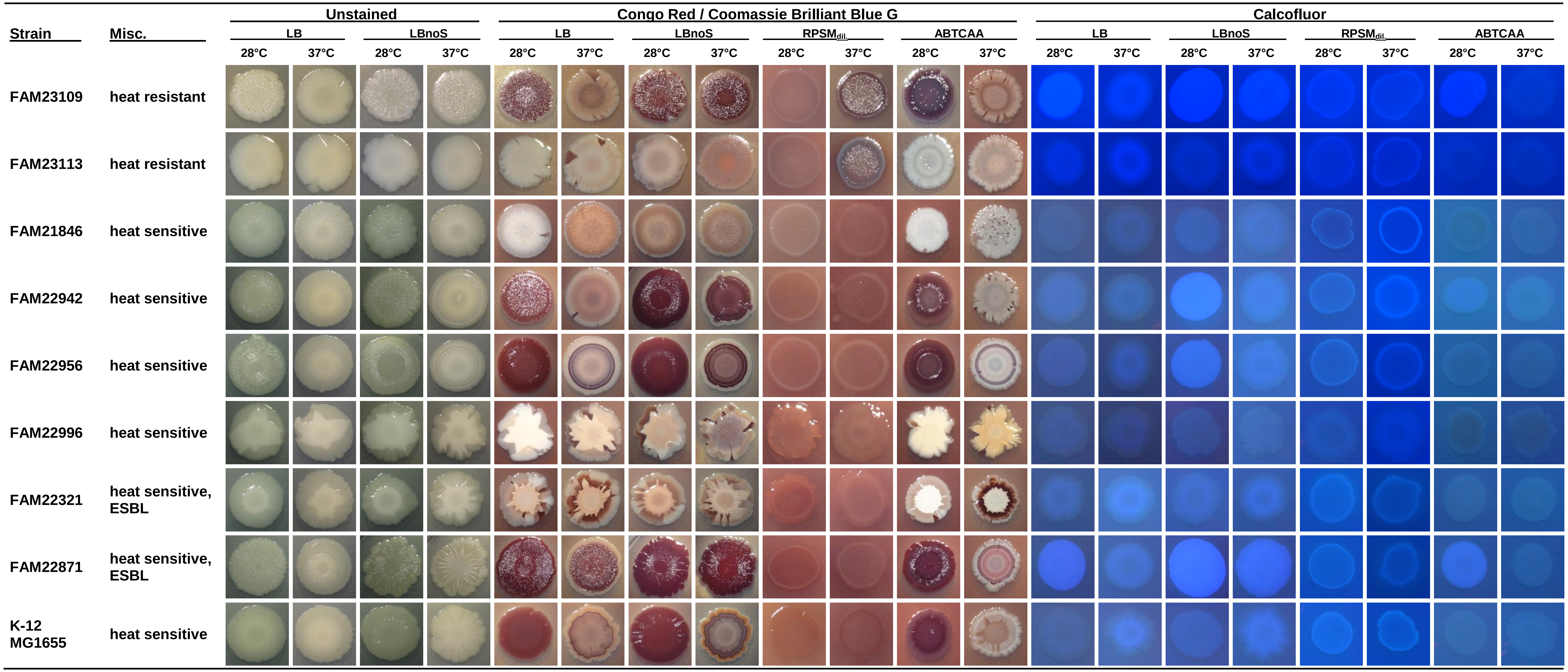


**FIG S1** Macrocolony assays of all 37 *E. coli* strains tested in this study. Unstained and Congo Red / Coomassie Brilliant Blue stained plates were incubated for 7d and Calcofluor stained plates for 3d at the indicated temperatures. LB: Luria-Bertani Lennox (10 g/L peptone, 5 g/L yeast extract, 5 g/L NaCl, pH 7.0), LBnoS: LB without addition of NaCl, ABTCAA: AB minimal media with 0.5% casamino acids as carbon source, $RPSM_{dil}$: reconstituted powdered skim milk (0.2%, wt/vol), ESBL: extended-spectrum β-lactamase producer. Congo red: 40µg/ml, Coomassie Brilliant Blue G: 20µg/ml, Calcofluor Fluorescent Brightener 28: 0.01%. All plates 1.2% (wt/vol) agar.
